# Artificial intelligence in odontology education

**DOI:** 10.3389/fdgth.2026.1965185

**Published:** 2026-10-27

**Authors:** María Belén Morales-Cevallos, Canva Byron Ma Lam, Kathy Violeta Serrano Avalos, Mónica Jimena Concha Guailla, Diego Fabian Vique Lopez

**Affiliations:** 1 Universidad Ecotec, Samborondón, Ecuador; 2 Universidad Católica Santiago de Guayaquil, Guayaquil, Ecuador; 3 Facultad de Salud Pública, Escuela Superior Politécnica de Chimborazo (ESPOCH), Riobamba, Ecuador; 4 Italian University Institute of Rosario (IUNIR), Rosario, Argentina

**Keywords:** AI, dental education, dental students, education, intelligent tutoring, learning, training, tutoring systems

## Abstract

**Introduction:**

Artificial intelligence (AI) is increasingly being explored in dental education as a tool to support teaching, learning, assessment, and clinical decision-making. This systematic review synthesized current evidence on the implementation and educational impact of AI technologies in undergraduate, postgraduate, and continuing dental education.

**Methodology:**

A PRISMA-guided systematic review was conducted using Scopus, PubMed, and IEEE Xplore. Studies published between 2020 and 2025 evaluating AI applications in dental education were identified using predefined PICO criteria. Educational outcomes included knowledge acquisition, diagnostic accuracy, clinical reasoning, procedural skills, learner engagement, and perceptions of AI-assisted learning.

**Results:**

The included literature reflects an expansion from machine learning, deep learning, and computer vision systems primarily evaluated for diagnostic support toward generative AI platforms, including large language models and retrieval-augmented generation systems. Across the included studies, AI applications were frequently associated with favorable diagnostic and examination performance and, in selected educational contexts, with supportive findings related to clinical reasoning, technical competency, feedback, and self-directed learning. However, the evidence was predominantly based on technical performance assessments and observational or cross-sectional designs.

**Discussion:**

AI is increasingly being explored as a potential complementary educational resource, with applications including interactive tutoring, adaptive learning, and support for clinical reasoning. Nevertheless, heterogeneous methodologies and limited longitudinal evidence indicate the need for standardized educational evaluation and responsible curricular integration.

**Conclusion:**

AI shows potential as a complementary resource in dental education. Its integration should remain pedagogically guided and ethically supervised while future research establishes its educational effectiveness through standardized and longitudinal evaluation.

## 1 Introduction

The landscape of modern healthcare is undergoing a profound paradigm shift driven by the rapid maturation and integration of artificial intelligence (1). Within the domain of dentistry (2), these technologies have transcended the realm of theoretical research to become increasingly ubiquitous tools that influence diagnostic precision (3), treatment planning, and clinical outcomes (3, 4). As these innovations migrate from experimental laboratories into the heart of clinical practice (5), the urgency to integrate artificial intelligence-related competencies into dental education has become a paramount concern for academic institutions globally (6). Dental schools are currently faced with the significant challenge of evolving their curricula to prepare the next generation of practitioners to interact (7), interpret (8), and critically evaluate the sophisticated systems that will define their professional careers (9). This systematic review is designed to synthesize the current evidence regarding the pedagogical strategies (10), technological applications (11), and ethical considerations that surround the implementation of artificial intelligence within the field of odontology education (12).

The traditional model of dental training (13), which has long been anchored in didactic learning and analog clinical simulation (14), is being rigorously challenged by the necessity for advanced digital fluency (15). The clinical practice of the current era is defined by augmented dentistry (16), where clinicians rely heavily on computer-aided design and manufacturing (17), complex three-dimensional imaging (16), and diagnostic aids powered by algorithms (17). Consequently, undergraduate and postgraduate curricula must pivot toward a robust (18), competency-based model that treats artificial intelligence literacy as a foundational skill (19), much like the proficiency required in traditional radiology or oral pathology (20). The tools currently making inroads into dental curricula include adaptive learning platforms that personalize the educational experience based on individual student performance data (21), as well as intelligent tutoring systems that provide real-time (22), objective feedback (23). These tools may provide a more standardized and continuous approach to feedback compared with the subjective and intermittent grading processes that have historically characterized preclinical exercises (24). Furthermore, the convergence of artificial intelligence with virtual and augmented reality has transformed the nature of simulation training (25). Unlike traditional phantom heads (26), these advanced systems allow for repetitive (5), high-fidelity practice where the technology provides immediate (2), quantitative analysis of manual dexterity (18), the geometry of tooth preparations (27), and the nuances of clinical decision-making (28).

Despite the widespread enthusiasm surrounding these technological advancements (29), the current body of literature remains fragmented and at times inconsistent (8). While numerous studies have extensively documented the technical capabilities of these systems in identifying dental pathologies such as caries or periodontal disease on radiographs (30), there is a notable and concerning paucity of rigorous (31), systematic evaluation regarding the actual educational efficacy of these tools (32). There is a persistent need to determine whether artificial intelligence-assisted training modules truly improve a student's long-term diagnostic accuracy when compared to traditional (33), human-led instruction (33). Moreover, it remains unclear to what extent an over-reliance on these automated systems might inadvertently impede the development of independent clinical judgment and diagnostic reasoning (14). A further complication is the ambiguity in the literature regarding the definition of artificial intelligence in educational contexts (34), as many studies conflate simple software algorithms with true machine learning or deep neural networks (35). This lack of standardized terminology hinders the ability of the academic community to compare educational interventions and assess their true impact on learning outcomes (1). This systematic review addresses these critical gaps by meticulously screening the existing body of knowledge and distinguishing between superficial technological adoption and genuine pedagogical transformation (22).

The integration of these technologies into the dental curriculum is also accompanied by substantial barriers that extend beyond the practical challenges of faculty development (36), infrastructure investment, and curriculum design (1). Deep-seated ethical concerns persist (37), particularly regarding the black-box nature of many algorithms that provide clinical results without clearly explaining their underlying rationale (37, 38). In an educational environment (27), the ability of a student to understand and articulate the logic behind a clinical finding is just as vital as the finding itself (39). If students are trained to accept the outputs of machines uncritically (40), they risk losing the foundational reasoning skills that are essential for safe and responsible professional practice (39). Additionally, the emergence of large language models and generative artificial intelligence in academic settings presents a dual-edged sword (41). While these tools can democratize access to information and streamline literature review processes (42), they also introduce significant risks regarding academic integrity (31), the potential for algorithmic bias (36), and the propagation of outdated or incorrect clinical information (43). Addressing these concerns requires a fundamental shift toward an educational framework that emphasizes a deep form of literacy—the ability to understand (44), critique (44), and ethically utilize these systems—rather than merely teaching the mechanics of the software (45).

This review is ultimately guided by the necessity of creating a structured, evidence-based approach to curricular reform. The primary goal is to determine the current state of evidence regarding the impact of these educational interventions on student learning, skill acquisition, and clinical decision-making. By analyzing studies published over the last decade, this work categorizes the evidence into key domains, including preclinical simulation, diagnostic training, performance assessment, and content creation. By synthesizing these diverse findings, this review serves as a roadmap for dental schools navigating the complex transition toward a fully integrated curriculum. It provides a vital resource for educators and policymakers seeking to design pedagogical strategies that are not only technologically advanced but also clinically responsible, ethically grounded, and focused on the core mission of producing competent, thinking, and compassionate dental professionals.

## 2 Methodology

This systematic review was conducted in accordance with the Preferred Reporting Items for Systematic Reviews and Meta-Analyses (PRISMA) 2020 statement (46), ensuring methodological transparency, reproducibility, and a rigorous approach to evidence synthesis, ensuring methodological transparency, reproducibility, and a rigorous approach to evidence synthesis. A structured review protocol was implemented to systematically identify, screen, evaluate, and synthesize published studies examining the integration of AI technologies into dental education. Following the PRISMA framework minimized the risk of selection bias while providing a transparent and reproducible process for study identification and inclusion.

The review focuses on the application of AI-driven technologies within undergraduate, graduate, and continuing dental education, encompassing a broad range of educational settings and learning modalities. Particular attention was given to AI methodologies, including machine learning, deep learning, generative artificial intelligence, computer vision, natural language processing, and intelligent tutoring systems, as well as their influence on educational outcomes, clinical decision-making, diagnostic training, and competency development in dentistry. This comprehensive approach enables the evaluation of both the technological characteristics of AI systems and their pedagogical effectiveness in supporting dental education.

To capture the diversity of available evidence, the review includes multiple study designs, such as randomized controlled trials, quasi-experimental investigations, observational studies, cohort studies, cross-sectional surveys, and mixed-methods research. These studies were synthesized to assess key educational outcomes, including knowledge acquisition, diagnostic accuracy, clinical reasoning, procedural skill development, learner engagement, educational efficiency, and perceptions of AI-assisted learning environments. Establishing a rigorous methodological framework is essential given the rapidly evolving and heterogeneous nature of research on artificial intelligence in dental education. Adherence to the PRISMA recommendations provides a robust foundation for generating reliable evidence, identifying current research trends and knowledge gaps, and supporting future evidence-based implementation of AI technologies within dental curricula and professional training programs.

### 2.1 PICO framework and selection criteria

The Population, Intervention, Comparison, and Outcome (PICO) framework was applied to formulate the guiding research question and establish clearly defined eligibility criteria for this systematic review (Table 1). This structured approach ensured that the review remained focused on the educational and technological implications of integrating artificial intelligence into odontology education. By using the PICO framework, studies could be systematically classified according to the target population, AI-based educational intervention, comparator conditions, and reported learning outcomes.

**Table 1:** PICO framework defining the core elements for the systematic review on artificial intelligence applications in odontology education.

	Element	Description
P	Population	Undergraduate dental students, postgraduate residents, dental faculty members, practicing dentists, and learners enrolled in continuing professional education programs in odontology.
I	Intervention	Educational strategies, simulation platforms, diagnostic training systems, virtual tutoring tools, and clinical decision-support resources incorporating artificial intelligence, including machine learning, deep learning, computer vision, natural language processing, and generative AI models.
C	Comparison	Conventional odontology education approaches, including lecture-based instruction, textbook-guided learning, traditional clinical supervision, non-AI digital resources, and standard simulation methods.
O	Outcome	Measurable educational outcomes, including knowledge acquisition, diagnostic accuracy, clinical reasoning, procedural skill development, learner engagement, academic performance, confidence, and perceptions toward AI-assisted dental education.

In this review, the population comprised dental students, residents, faculty members, and dental professionals engaged in undergraduate, postgraduate, or continuing odontology education. The intervention included artificial intelligence–based tools such as machine learning models, deep learning systems, computer-assisted diagnostic platforms, virtual simulation environments, intelligent tutoring systems, and generative AI applications. Comparators included traditional teaching strategies, conventional digital learning methods, or non-AI-assisted educational approaches. Outcomes of interest included knowledge acquisition, diagnostic accuracy, clinical reasoning, procedural skill development, learner engagement, educational efficiency, and perceptions of AI-supported learning. This framework provided a rigorous basis for selecting relevant studies and synthesizing evidence on the reported associations and potential educational relevance of AI technologies for teaching, learning, and competency development in odontology education.

For eligibility and evidence synthesis, studies were additionally classified according to their educational or training relevance. Studies directly involving learners, educational interventions, training activities, examinations, educational assessment, or continuing professional development were considered direct educational evidence. Clinical or diagnostic AI studies without a direct learner intervention were retained only when the evaluated task had explicit relevance to dental training competencies, including diagnostic reasoning, radiographic interpretation, clinical decision-making, or treatment planning. These studies were classified as potential educational applications and were not interpreted as direct evidence of educational effectiveness.

The PICO framework was used to refine the primary research question guiding this systematic review:

“How have artificial intelligence-based tools been implemented and assessed in odontology education in relation to teaching strategies, student learning outcomes, clinical reasoning, diagnostic training, and competency development?”

This question emphasizes the inclusion of studies reporting empirical evidence rather than purely conceptual or theoretical discussions. Accordingly, the eligibility criteria prioritized investigations demonstrating measurable effects on educational performance, diagnostic accuracy, clinical skill acquisition, learner engagement, instructional efficiency, or perceptions of AI-assisted learning within dental education settings.

This review specifically considered studies published between 2020 and 2025, a period marked by rapid advances in machine learning, deep learning, generative artificial intelligence, and adaptive educational technologies. This time frame was selected to ensure the relevance and currency of the evidence, as earlier AI systems may not reflect the complexity, interactivity, or personalization capabilities of contemporary AI-driven tools used in odontology education.

The systematic identification of relevant literature was conducted using a multi-stage search strategy designed to capture the interdisciplinary scope of artificial intelligence in odontology education. To ensure broad coverage of both technological innovations and educational applications in dentistry, three major academic databases were selected: Scopus, PubMed, and IEEE Xplore. These databases provide extensive coverage of dental sciences, health professions education, computer science, biomedical engineering, and applied artificial intelligence, allowing for a comprehensive examination of the current evidence on AI-supported dental education.

A structured Boolean search strategy was developed to identify empirical studies with direct relevance to the research objective. The search query shown in Table 2 was applied consistently across the selected databases to maintain methodological uniformity. This combination of terms was intended to retrieve studies addressing the integration of AI-based tools into dental teaching and learning environments, rather than general applications of digital technologies in dentistry.

**Table 2:** The literature search strategy and the number of records retrieved from each electronic database are summarized.

Database	Query	Results
Scopus	( “artificial intelligence” OR “AI” ) AND ( “dentistry” OR “dental education” OR “oral health” OR “dental school” OR “dental students” OR “dental curriculum” ) AND ( “education” OR “learning” OR “teaching” OR “training” OR “competency” OR “clinical education” )	1,084
PubMed		42
IEEE Xplore		104

The inclusion of dental education-related terms ensured that the search focused on studies examining AI applications in academic, clinical, and simulation-based odontological training. This strategy allowed the review to identify research evaluating AI-assisted learning, diagnostic training, clinical decision-making, procedural skill development, student performance, and educational efficiency within odontology education.

For study classification and evidence synthesis, educational relevance was additionally considered according to the primary purpose and context of each investigation. Studies directly involving learners, educational interventions, training activities, examinations, learner assessment, or continuing professional development were classified as providing direct educational or training evidence. Studies primarily evaluating the clinical or diagnostic performance of AI systems were considered separately as providing indirect or potential educational evidence when their findings had explicit relevance to diagnostic training, clinical decision-making, competency development, or future educational application. Accordingly, diagnostic accuracy or clinical performance was interpreted as an educational outcome only when assessed within a learner, training, or educational evaluation context; technical AI performance alone was not considered evidence of educational effectiveness. Importantly, competency-linked clinical studies were not retained on the basis of dental context alone; eligibility required that the evaluated task correspond to a defined component of dental training, such as diagnostic reasoning, radiographic interpretation, disease recognition, treatment planning, clinical decision-making, or critical evaluation of AI-generated information

The search was designed to identify studies examining the implementation, evaluation, and educational impact of artificial intelligence-based tools in odontology education. Search terms combined concepts related to artificial intelligence, dentistry, dental education, oral health, dental students, clinical training, learning, teaching, competency development, and curriculum design.

The initial search results reflected the disciplinary scope and indexing characteristics of each database. Scopus produced the largest number of records, highlighting its relevance as a broad multidisciplinary database for identifying literature related to artificial intelligence and dental education. PubMed contributed studies mainly focused on biomedical, clinical, and educational aspects of dentistry, whereas IEEE Xplore provided publications primarily associated with the technical development, digital architecture, and computational implementation of AI-based educational tools.

The search was limited to studies published between 2020 and 2025 to capture recent advances in machine learning, deep learning, generative artificial intelligence, computer vision, intelligent tutoring systems, and adaptive learning technologies applied to odontology education. This time frame was selected to ensure that the review reflects contemporary AI applications with potential relevance for dental training, diagnostic education, simulation-based learning, and clinical competency development.

The search strategy prioritized peer-reviewed journal articles and full conference proceedings to ensure the inclusion of methodologically reliable and academically relevant evidence. Duplicate records retrieved across the selected databases were identified and removed during the initial screening phase. This process established the empirical basis for evaluating how artificial intelligence is being integrated into dental education and how these technologies influence student learning, diagnostic performance, clinical reasoning, procedural training, and educational efficiency.

By narrowing the scope to the intersection of artificial intelligence and odontology education, this review pursued three main objectives: first, to identify studies with clearly defined educational outcomes related to dental students, trainees, or professionals; second, to improve retrieval precision by excluding studies focused solely on general clinical AI without either a direct educational component or an explicitly identifiable relevance to dental training, assessment, competency development, or educational application; and third, to maintain methodological consistency by aligning the selection process with the predefined PICO-based eligibility criteria (47, 48).

The annual distribution of publications demonstrates a consistent and substantial increase in research related to AI in odontology education between 2020 and 2025 (Figure 1). Across all years, Scopus yielded the highest number of records, indicating its broad multidisciplinary coverage of AI applications in dental education, while PubMed and IEEE Xplore contributed a smaller but complementary body of literature from biomedical and engineering perspectives. During the early years of the study period, publication output was relatively limited. In 2020, the literature search identified 21 publications in Scopus, 10 in IEEE Xplore, and 1 in PubMed, suggesting that research on AI-assisted dental education was still emerging. In 2021, the number of publications increased to 53 in Scopus, whereas PubMed and IEEE Xplore retrieved 6 and 4 publications, respectively. This upward trend continued in 2022, when Scopus reached 88 publications, while IEEE Xplore and PubMed identified 16 and 3 publications, respectively.

**Figure 1:**
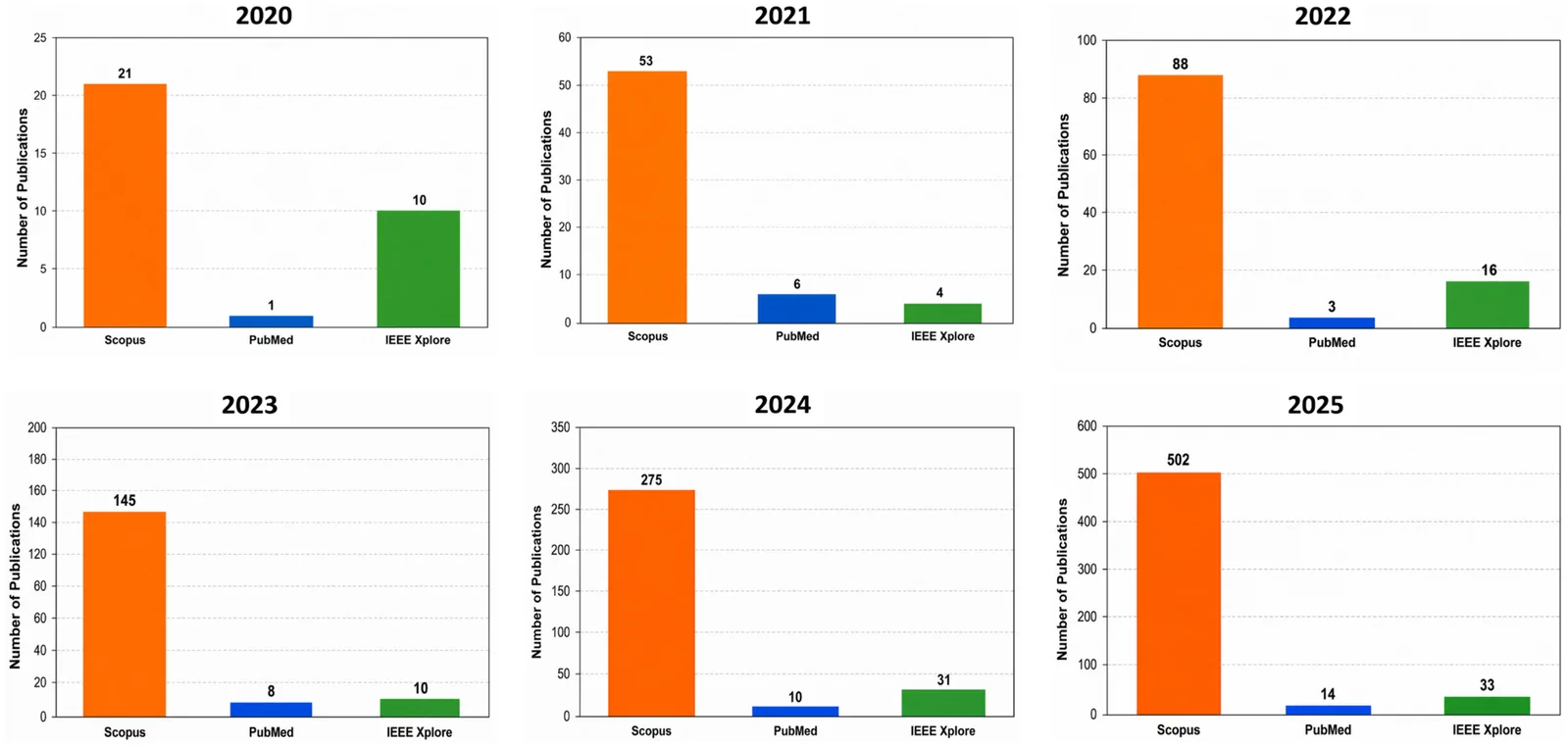
Annual number of publications indexed in scopus, pubMed, and IEEE xplore from 2020 to 2025 related to artificial intelligence tools in odontology education.

A marked acceleration in research productivity became evident beginning in 2023. Scopus nearly doubled its output to 145 publications, whereas IEEE Xplore and PubMed identified 10 and 8 studies, respectively. The increase continued in 2024, with 275 publications indexed in Scopus, accompanied by 31 publications in IEEE Xplore and 10 in PubMed, reflecting growing interest in AI-driven educational technologies and their application to dental training. The most pronounced growth occurred in 2025, when Scopus indexed 502 publications, representing approximately a 24-fold increase compared with 2020. During the same year, IEEE Xplore reported 33 publications, while PubMed reached 14 publications, indicating a gradual expansion of clinically oriented and technology-focused research.

### 2.2 Identification

The identification and selection of relevant studies for this systematic review were performed in accordance with the PRISMA guidelines (46). Figure 2 illustrates the sequential stages of the screening process, showing the flow of records from the initial database search to the final inclusion of studies eligible for systematic analysis in the context of artificial intelligence applications in odontology education.

**Figure 2:**
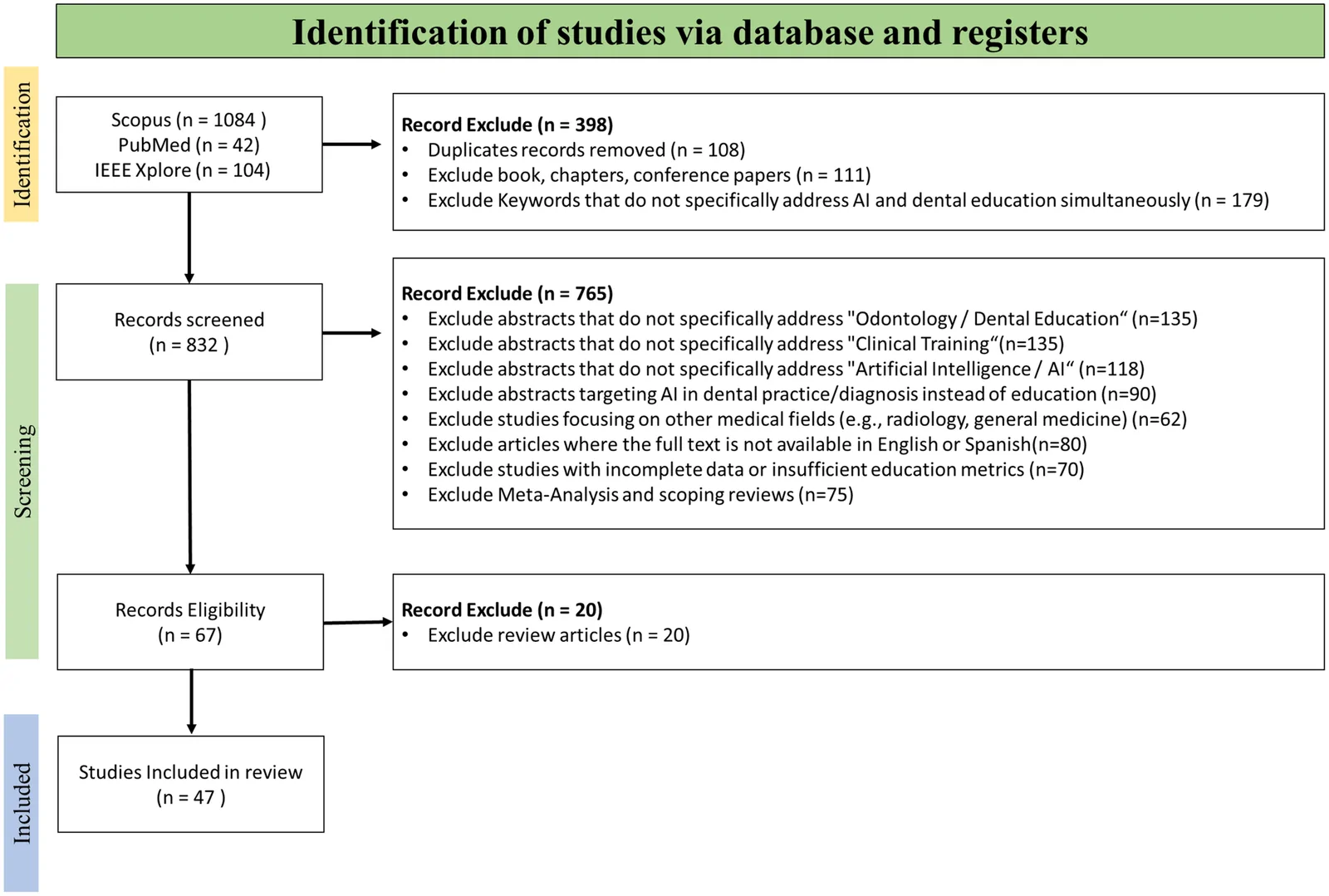
PRISMA flow diagram illustrating the identification, screening, eligibility assessment, and inclusion of studies in this systematic review. The diagram was created by the authors in accordance with the PRISMA 2020 statement (46).

The identification phase consisted of a comprehensive search across three electronic databases: Scopus (*n* = 1084), PubMed (*n* = 42), and IEEE Xplore (*n* = 104). This search retrieved a total of 1,230 records. The variation in the number of records obtained from each database reflects their different disciplinary scopes, with Scopus providing the broadest coverage of interdisciplinary research related to artificial intelligence, dental education, and oral health sciences. To ensure the accuracy and integrity of the dataset, duplicate records, noneligible publication types, and records that did not simultaneously address artificial intelligence and dental education were removed during the initial selection process. After these exclusions, 832 records were retained for title and abstract screening.

### 2.3 Screened

During the screening phase, 832 records were assessed according to title, abstract, keywords, and thematic relevance. This stage was designed to identify studies that explicitly addressed the intersection between artificial intelligence and odontology or dental education. A total of 765 records were excluded because they did not meet the predefined screening criteria. These exclusions included abstracts that did not specifically address odontology or dental education (*n* = 135), clinical training (*n* = 135), or artificial intelligence/AI (*n* = 118). Additional records were excluded because they focused on AI in dental practice or diagnosis rather than education (*n* = 90), addressed other medical fields such as radiology or general medicine (*n* = 62), were not available in English or Spanish (*n* = 80), reported incomplete data or insufficient educational outcome metrics (*n* = 70), or corresponded to meta-analyses and scoping reviews (*n* = 75).

### 2.4 Eligibility

The eligibility phase involved a full-text evaluation of the 67 remaining articles to determine their alignment with the objectives of this systematic review. At this stage, studies were assessed based on their methodological quality, relevance to odontology education, and availability of empirical data related to educational outcomes. Review articles and papers lacking original evidence on AI-assisted learning, clinical training, diagnostic education, or competency development in dentistry were excluded. After full-text assessment, 47 studies met the inclusion criteria.

### 2.5 Inclusion

The final inclusion phase resulted in 47 studies being included in the systematic review. These articles represented original empirical research evaluating the implementation or impact of artificial intelligence tools in dental education. The included studies provided evidence on outcomes such as student learning, diagnostic accuracy, clinical reasoning, procedural training, educational efficiency, and perceptions of AI-assisted learning environments. This final sample established the evidence base for analyzing how artificial intelligence is being integrated into odontology education and their potential relevance to the development of clinical and academic competencies in dental training.

## 3 Results

### 3.1 Impact and maturity of artificial intelligence applications in dental education

The evidence summarized in Table 3 indicates collective investigation and application of AI across contexts relevant to dental education. Many included studies reported favorable findings, although the nature of these outcomes varied considerably and frequently reflected technical or diagnostic performance rather than direct educational effectiveness. Collectively, the findings suggest that AI may have potential to support diagnostic reasoning, personalized learning, assessment, and competency-based training, but further educational evaluation is required.

**Table 3:** Summary of the educational characteristics of studies evaluating artificial intelligence in dental education, including study aims, educational level, study design, AI technology, reported educational effect, outcome variable, and stage of AI implementation.

Reference	Study Aim	AI Technology	Effect	Variable	Stage	Educational/Training Relevance
Pawar et al. (73)	To evaluate whether machine-learning algorithms can predict tooth loss/prognosis from patient systemic status and dental parameters such as age, diabetes, hypertension, tooth mobility, oral hygiene, furcation, treatment modality, and prognosis.	Naive Bayes, AdaBoost, Random Forest, Neural Network, decision trees/support vector approaches via Orange	Positive	Performance	Applied	Indirect competency-linked educational relevance—prognostic reasoning and clinical decision-making
Durmazpinar, et al. (49)	To compare ChatGPT-4o's diagnostic accuracy in endodontic cases with 3rd-year and 5th-year dental students using multimodal clinical questions based on radiographs, photographs, and patient histories.	ChatGPT-4o	Positive	Performance	Applied	Learner assessment/educational evaluation—AI performance compared with dental students in diagnostic cases
Vazirizadeh; et al. (59)	To develop and evaluate an attention-based residual CNN for automatic detection of lip competence/incompetence on orthodontic profile and frontal photographs.	Attention-based residual convolutional neural network (ARN-CNN) with image preprocessing, augmentation, Grad-CAM visualization	Positive	Performance	Development	Indirect competency-linked educational relevance—orthodontic visual assessment and diagnostic interpretation
Abou-Bakr; et al. (69)	To investigate postgraduate dental students' perceptions, usage, and concerns regarding AI applications in dental education and practice.	General AI applications, including AI-based software and large language models	Positive	Engagement	Initially	Educational perception/CPD—postgraduate students' AI use, perceptions, and training needs
León de la Fuente; et al. (85)	To evaluate the reliability and repeatability of ChatGPT-4 and ChatGPT-4o when answering practitioner-level endodontic questions and to consider implications for AI-enhanced clinical learning.	ChatGPT-4 and ChatGPT-4o	Increased	Performance	Applied	Indirect competency-linked educational relevance—endodontic knowledge and clinical reasoning support
Çiçek; et al. (60)	To develop and evaluate a deep learning model for detecting and segmenting dental plaque in young permanent teeth and compare its performance with experienced pediatric dentists.	Deep learning using U-Net-based architectures; best-performing U-Net Transformer	Positive	Performance	Development	Indirect competency-linked educational relevance—plaque recognition and preventive diagnostic competency
Yurt; et al. (50)	To compare the accuracy of third- and fifth-year dental students and AI chatbots in answering multiple-choice questions on pulpal and periapical disease diagnosis based on AAE guidelines.	NLP-based generative AI/LLMs: ChatGPT-3.5, ChatGPT-4o, Gemini, Microsoft Copilot	Positive	Performance	Applied	Learner assessment/educational evaluation—comparison with dental students on guideline-based diagnostic questions
Shukla; et al. (70)	To assess Indian dental students' knowledge, attitudes, perceptions, and ethical awareness regarding the integration of AI in dentistry.	Artificial intelligence in dental diagnostics, prognosis, treatment planning, radiographic interpretation, and ethics	Positive	Engagement	Initially	Educational perception/AI literacy—students' knowledge, attitudes, ethical awareness, and educational needs
Fukuda; et al. (51)	To evaluate the accuracy and response capability of ChatGPT-4o when solving Japanese National Dental Technician Examination questions and assess its potential as a supportive learning and assessment tool.	ChatGPT-4o multimodal/generative LLM	Positive	Performance	Applied	Learner assessment/educational evaluation—dental examination performance and potential learning support
Sevimli; et al. (74)	To compare diagnostic accuracy of fourth-year and final-year undergraduate dental students with an AI application when examining panoramic radiographs.	Machine learning/computer vision for panoramic radiograph analysis	Positive	Performance	Applied	Learner assessment/clinical diagnostic training—student radiographic interpretation compared with AI
Xu; et al. (61)	To develop and evaluate a RAG-enhanced LLM framework integrating domain-specific multimodal knowledge for AI-driven endodontic education.	Retrieval-Augmented Generation with large language models and multimodal knowledge base	Positive	Skill development	Development	Direct educational intervention—RAG-enhanced system specifically developed for endodontic education
Caponio; et al. (93)	To assess whether operator experience in a postgraduate educational setting, along with patient, implant, and surgical risk factors, influenced dental implant failure.	Not applicable in study; artificial intelligence mentioned as potential future curriculum tool	Positive	Performance	Applied	Clinical training relevance—postgraduate operator experience and clinical competency development
Suraiya, A.; et al. (62)	To evaluate the efficiency of a CNN algorithm in detecting alveolar bone loss on panoramic radiographs and classifying periodontal bone-loss versus healthy periodontium cases.	VGG-16 convolutional neural network; transfer learning	Positive	Performance	Development	Indirect competency-linked educational relevance—periodontal radiographic interpretation and bone-loss recognition
Abdulmajeed. et al. (71)	To assess knowledge, awareness, and clinical utilization of digital endodontic technologies among dental professionals in southern Saudi Arabia and compare adoption between specialists, general practitioners, and interns.	Digital radiography, CBCT, electronic apex locators, guided endodontics, 3D printing, AI-assisted diagnosis	Positive	Engagement	Initially	Educational perception/CPD—professional knowledge, technology awareness, adoption, and training needs
Ji; et al. (52)	To evaluate ChatGPT-4 performance in answering oral and maxillofacial disease questions using standard prompting and Chain-of-Thought prompting, and to assess its capabilities, potential, and limitations.	ChatGPT-4 with and without Chain-of-Thought prompting	Positive	Performance	Applied	Indirect competency-linked educational relevance—oral/maxillofacial knowledge, reasoning, and AI response appraisal
Alnsour; et al. (94)	To evaluate ChatGPT's effectiveness in answering public questions about the effects of smoking on oral health, focusing on usefulness, quality, reliability, readability, understandability, and actionability.	ChatGPT 3.5; natural language generation based on large language model technology	Positive	Performance	Applied	Indirect competency-linked educational relevance—oral-health information appraisal, patient communication, and AI literacy
Bostanci et al. (63)	To develop and evaluate a convolutional neural network for automatic assessment of open versus closed apical status in permanent first molars using CBCT-derived pseudopanoramic region-of-interest images.	Convolutional neural network (CNN) trained on CBCT-derived pseudopanoramic images	Positive	Performance	Development	Indirect competency-linked educational relevance—radiographic interpretation and apical-status diagnostic competency
Felemban et al. (53)	To compare the accuracy, consistency, and response performance of ChatGPT-3.5, ChatGPT-4, and ChatGPT-4 Turbo on validated MCQs in oral/maxillofacial pathology and oral radiology.	ChatGPT-3.5, ChatGPT-4, ChatGPT-4 Turbo	Positive	Performance	Applied	Learner assessment/educational evaluation—AI performance on validated oral pathology and radiology MCQs
Ghasemian et al. (72)	To assess Iranian dental students' attitudes, perceptions, and perceived educational needs regarding AI in dentistry and its future role in diagnosis, treatment planning, and practice.	Validated questionnaire assessing attitudes toward AI applications in dentistry	Positive	Engagement	Initially	Educational perception/AI literacy—students' attitudes and perceived educational needs regarding AI
Ghorbani et al. (64)	To develop and evaluate an automated AI system for detecting and numbering primary and permanent teeth on maxillary and mandibular occlusal photographs.	CNN using YOLOv8; two-model pipeline for tooth detection, bounding boxes, localization, classification, and numbering	Positive	Skill development	Development	Indirect competency-linked educational relevance—tooth identification, classification, and anatomical recognition
Kandemir; et al. (75)	To compare the accuracy and coverage of ChatGPT-4, Claude, Gemini Advanced, and DeepSeek-R1 on Turkish Dental Specialty Examination periodontology questions and by question type.	Natural language processing; generative AI; ChatGPT-4, Claude, Gemini Advanced, DeepSeek-R1	Positive	Performance	Applied	Learner assessment/educational evaluation—AI performance on dental specialty examination questions
Eraslan; et al. (76)	To evaluate the accuracy of five AI chatbots in answering prosthodontics questions from the Dentistry Specialization Residency Examination across seven subtopics.	Natural language processing; generative AI chatbots	Positive	Performance	Applied	Learner assessment/educational evaluation—prosthodontic residency examination performance
Çeki˙ç; et al. (54)	To evaluate and compare eight large language models in answering standard MCQs and combination-type MCQs from Turkish national endodontic specialty examinations.	Large language models; natural language processing; deep learning neural networks	Positive	Performance	Applied	Learner assessment/educational evaluation—endodontic specialty examination performance
Sabri; et al. (55)	To quantify and compare the accuracy of GPT-4, GPT-3.5, and Google Gemini against periodontal residents on American Academy of Periodontology in-service examination questions.	Large language models; natural language processing	Positive	Performance	Applied	Learner assessment/educational evaluation—AI performance compared with periodontal residents in an in-service examination
Ehrensperger; et al. (88)	To determine how well gingivitis is visually detectable using an easy-to-use intraoral camera application with a machine-learning-based filter compared with clinical bleeding-on-probing and dentist visual assessment.	Machine-learning image filter applied to standardized intraoral photographs	Negative	Performance	Applied	Indirect competency-linked educational relevance—gingivitis recognition and visual diagnostic interpretation
Sabbagh; et al. (80)	Evaluate the effectiveness and student acceptance of the Munich Cephalometric Application for Training (MCAT), an AI-supported tool for cephalometric tracing in orthodontic education.	Deep-learning-based automated cephalometric landmark detection with reference-point feedback	Positive	Skill development	Applied	Direct educational intervention—AI-supported cephalometric training and student acceptance
Lin; et al. (81)	Integrate digital and AI-assisted cephalometric training into preclinical orthodontic education and evaluate whether it improves landmarking accuracy and reduces performance differences among students.	Computer-assisted digital cephalometric scoring with AI-assisted cephalometric analysis	Increased	Skill development	Applied	Direct educational intervention—AI-assisted preclinical cephalometric training and learner performance
Erkal; et al. (89)	Assess whether advanced large language models can match expert endodontic reasoning in diagnosing and managing dens invaginatus cases using periapical radiographs with clinical vignettes.	Multimodal LLMs: GPT-4o, gpt-4o-mini, o1, o1-pro, Claude 3.5 Sonnet, and Microsoft Copilot	Negative	Performance	Applied	Indirect competency-linked educational relevance—endodontic diagnostic reasoning and case-management competency
Kaygısız,; et al. (65)	To develop and evaluate an AI-based system for detecting and classifying jaw cysts on panoramic radiographs using YOLO v11.	YOLO v11 Small deep learning object-detection model	Positive	Performance	Development	Indirect competency-linked educational relevance—radiographic recognition and differential classification of jaw cysts
Al-Jewari; et al. (95)	To detect dry socket development after lower molar extraction in patients with predisposing factors and examine associations with those factors.	Not applicable	Positive	Skill development	Applied	Clinical training relevance—postoperative complication recognition and risk-factor assessment
Zheng; et al. (86)	To develop and evaluate a deep learning method for automatically restoring missing regions in intraoral 3D point clouds to improve digital orthodontic workflows.	Point Fractal Network (PF-Net) deep learning architecture with GAN-related completion framework	Increased	Performance	Development	Indirect competency-linked educational relevance—digital orthodontic workflow and 3D data interpretation competency
Lafourcade; et al. (56)	To evaluate and compare LLM performance in restorative dentistry and endodontics using accuracy, consistency, repeatability, robustness, and contextual understanding on MCQs and definitions from national educational archives.	Natural language processing; large language models; context-based prompting/RAG-style educational content support	Positive	Performance	Applied	Learner assessment/educational evaluation—LLM performance using educational MCQs and curriculum-derived content
Keskin; et al. (84)	To assess and compare the radiographic diagnostic accuracy of undergraduate dental students, endodontics residency students, endodontists, and an AI application for caries, dislodged restorations, and periapical status.	AI-based radiographic image analysis; machine learning/deep learning diagnostic support	Positive	Performance	Applied	Learner assessment/clinical diagnostic training—radiographic performance compared across students, residents, specialists, and AI
Simanoglu; et al. (77)	To assess and compare ChatGPT-4.0 and Gemini Advanced performance on the 2020 and 2021 Turkish Dental Specialty Examination (DUS) and compare their scores with top human performers.	Large language models; natural language processing; AI-powered chatbots	Positive	Performance	Applied	Learner assessment/educational evaluation—AI performance on national dental specialty examinations compared with human performance
Kumaralingam; et al. (66)	To develop OralSAM, an end-to-end machine-learning system for automated segmentation and quantitative assessment of periodontal structures in intraoral ultrasound videos, including alveolar bone level, gingival thickness, and alveolar bone thickness.	Machine learning; deep-learning segmentation network; morphological analysis pipeline	Positive	Performance	Development	Indirect competency-linked educational relevance—periodontal image interpretation and quantitative assessment competency
Marwaha; et al. (92)	To create a fully automated deep-learning pipeline for segmenting carious lesions and classifying RVG images into no caries, enamel caries, and dentin caries.	U-Net for pixel-wise lesion segmentation; ResNet-50 convolutional neural network for multiclass classification	Positive	Performance	Development	Indirect competency-linked educational relevance—radiographic caries recognition and diagnostic classification
Büyüközer; et al. (90)	To evaluate the reliability and accuracy of AI-generated information on regenerative endodontic procedures across four AI models, two languages, and prompt/no-prompt conditions compared with clinical guidelines.	ChatGPT-4o, Claude 3.5 Sonnet, Grok 2, Gemini 2.0 Advanced	Negative	Performance	Applied	Indirect competency-linked educational relevance—critical appraisal of AI-generated endodontic information against clinical guidelines
Yu; et al. (82)	Develop and evaluate an automatic 2.5D Attention U-Net system for accurate 3D enamel segmentation, visualization, and thickness measurement from clinical CBCT data.	2.5D Attention U-Net deep learning segmentation with 3D reconstruction and enamel-thickness calculation	Positive	Skill development	Development	Indirect competency-linked educational relevance—CBCT interpretation, enamel visualization, and anatomical assessment
Jalali; et al. (57)	Assess the accuracy and reasoning quality of seven AI chatbots when answering 100 board-style endodontic multiple-choice questions.	Gemini Advanced, Gemini, Microsoft Copilot, GPT-3.5, GPT-4o, GPT-4.0, Claude 3.5 Sonnet	Positive	Performance	Applied	Learner assessment/educational evaluation—board-style endodontic knowledge and reasoning assessment
Tosun; et al. (78)	Evaluate seven AI-based chatbots on 128 prosthodontics multiple-choice questions from Turkey's Dental Specialty Exam and compare performance by question type.	ChatGPT-3.5, ChatGPT-4, Gemini, Gemini Advanced, Claude AI, Microsoft Copilot, Smodin AI	Positive	Performance	Applied	Learner assessment/educational evaluation—prosthodontic specialty examination and case-based reasoning assessment
Slashcheva; et al. (83)	To survey dental providers and patients about use of AI-enhanced radiographs in diagnostic and patient-education processes.	AI software annotating carious lesions and periodontal measurements on dental radiographs	Positive	Engagement	Applied	Clinical training/patient-education application—AI-assisted radiographic interpretation and patient communication
Camlet; et al. (87)	To assess effectiveness of ChatGPT-3.5 and ChatGPT-4 in answering periodontology specialization examination questions in Polish and English.	ChatGPT-3.5 and ChatGPT-4; GPT transformer-based language models	increased	Performance	Applied	Learner assessment/educational evaluation—periodontology specialty examination performance
Enkvetchakul; et al. (67)	To develop and validate an advanced AI model for classifying root dental diseases in x-rays using ensemble deep learning and metaheuristic optimization.	Ensemble CNNs, np-AMIS image augmentation, pop-AMIS decision fusion.	Positive	Performance	Development	Indirect competency-linked educational relevance—radiographic disease classification and diagnostic decision support
Hong; et al. [92]	Develop and validate ENPAT, a YOLOv8-based non-invasive periodontal assessment tool for screening anterior esthetic-zone periodontal conditions from intraoral photographs.	YOLOv8s-seg; YOLOv8-Ghost; ResNet-50 baseline	Positive	Performance	Development	Clinical training application—AI-supported periodontal assessment and improved junior-dentist agreement with experts
Khurshid; et al. (68)	Develop and compare deep learning models for automated detection of dental restorations and prostheses in panoramic radiographs using a multi-regional dataset.	YOLOv11; Faster R-CNN; Vision Transformer (ViT)	Positive	Performance	Development	Indirect competency-linked educational relevance—radiographic recognition of restorations and prostheses
Çakmak; et al. (58)	Evaluate AI-powered chatbots' accuracy on orthodontic Dental Specialty Exam questions and assess the reliability of their cited information sources.	ChatGPT-5; Claude 3.7; Copilot	Positive	Performance	Applied	Learner assessment/educational evaluation—orthodontic specialty examination performance and source-evaluation competency
İşisağ; et al. (91)	To evaluate ChatGPT-4.0 effectiveness in planning tooth-supported fixed prostheses by examining accuracy, repeatability, and consistency of responses across simple.	ChatGPT-4.0; natural language processing; large language model	Positive	Performance	Applied	Indirect competency-linked educational relevance—prosthodontic treatment planning and clinical reasoning competency

ARN-CNN, attention-based residual convolutional neural network; CBCT, cone-beam computed tomography; CNN, convolutional neural network; CPD, continuing professional development; Grad-CAM, gradient-weighted class activation mapping; LLM, large language model; NLP, natural language processing; PF-Net, point fractal network; RAG, retrieval-augmented generation; ViT, vision transformer; YOLO, you only look once.

Despite these encouraging findings, several research gaps remain. Most studies employed observational or cross-sectional designs, limiting conclusions regarding long-term educational effectiveness and knowledge retention. Well-designed randomized controlled educational trials are needed to compare AI-assisted instruction with traditional teaching methods using standardized educational outcomes. Furthermore, future investigations should evaluate cost-effectiveness, curriculum integration strategies, faculty readiness, and institutional implementation frameworks.

Ethical considerations also require greater attention. Dental schools must establish policies addressing algorithm transparency, data protection, academic integrity, and responsible AI use. Faculty development programs will be essential to ensure educators possess the digital competencies necessary to critically evaluate AI-generated information and effectively integrate these technologies into teaching. Finally, future systematic reviews should examine AI applications across individual dental specialties to identify discipline-specific educational benefits and limitations.

As summarized in Table 1 and illustrated in Figure 3, most of the included studies (29, 47) were classified within the Applied stage, followed by the Development stage (14, 47), whereas only four studies remained at the Initially stage. This distribution suggests that many of the AI technologies represented in the included literature have progressed from conceptual exploration to applied evaluation. But, classification as ‘Applied' does not necessarily indicate established curricular integration or demonstrated educational effectiveness.

**Figure 3:**
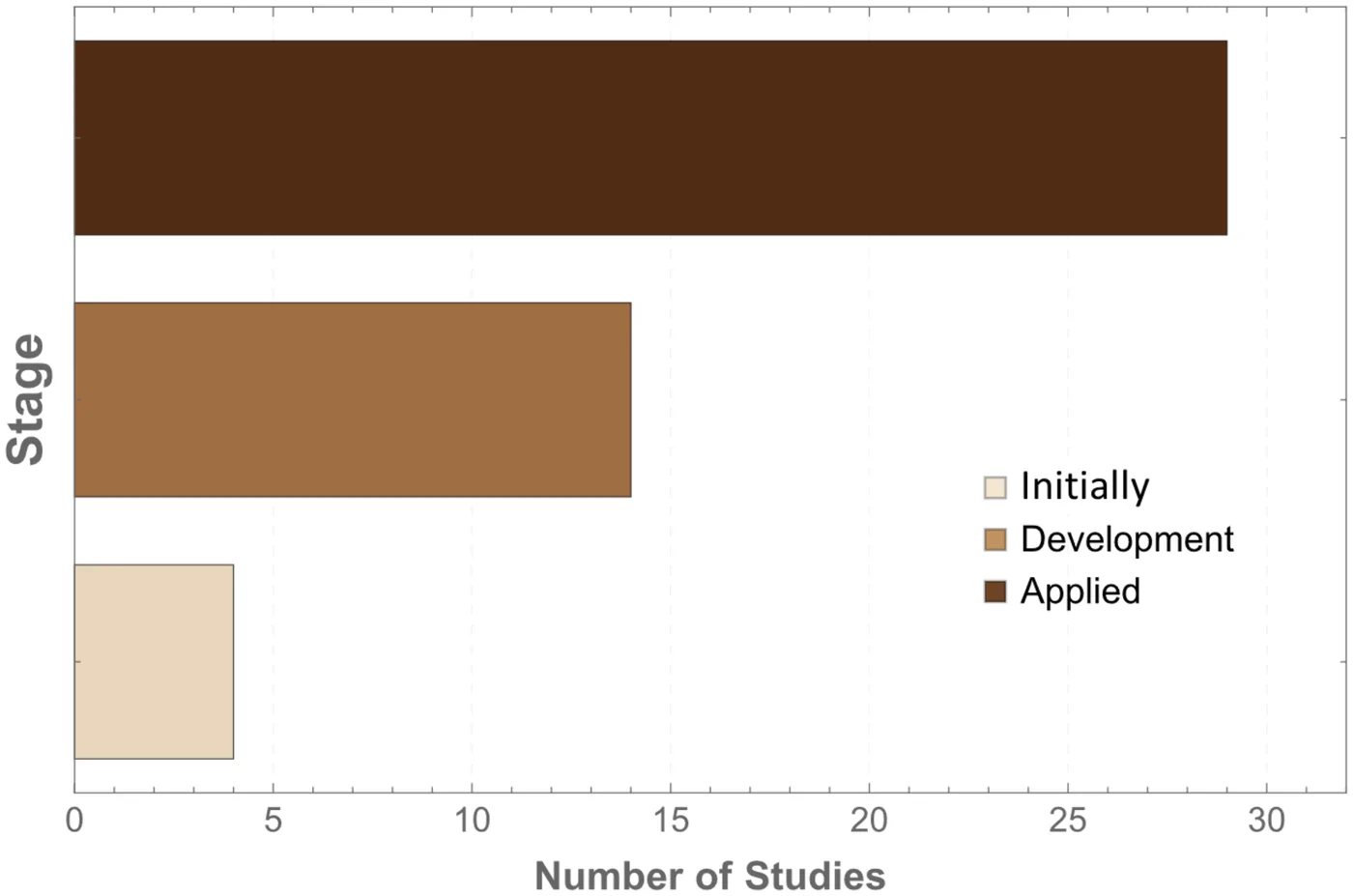
Distribution of included studies according to the stage of artificial intelligence implementation in dental education (initially, development, and applied).

The predominance of studies in the Applied stage reflects the growing availability of mature AI technologies, including LLMs, convolutional neural networks (CNNs), computer vision systems, and retrieval-augmented generation (RAG) frameworks (49–58). These technologies have been evaluated under authentic educational conditions, including undergraduate teaching, postgraduate training, specialty examination preparation, radiographic interpretation, and clinical decision-making. In contrast, studies classified as Development primarily focused on designing and validating AI algorithms for diagnostic support, image segmentation, automated landmark detection, and educational software (59–68). Meanwhile, studies in the Initially stage mainly investigated students' awareness, attitudes, ethical concerns, and readiness to adopt AI within dental curricula (69–72). Collectively, these findings indicate that dental education has entered a phase characterized by implementation and optimization rather than preliminary exploration.

As presented in Figure 4, performance was evaluated in 36 studies, whereas skill development and engagement were investigated in only 6 and 5 studies, respectively. This imbalance indicates that current research primarily focuses on evaluating AI-associated performance in measurable academic and clinical tasks. Most performance-oriented studies demonstrated that AI systems achieved diagnostic accuracy comparable to or exceeding that of undergraduate students, postgraduate trainees, and, in selected tasks, experienced clinicians (49–58, 73–78). Large language models showed strong performance in answering board-style examination questions across endodontics, prosthodontics, orthodontics, periodontology, and oral pathology, suggesting their potential as intelligent tutoring systems and examination support tools (53–57, 75–78). Likewise, computer vision algorithms demonstrated excellent capabilities in radiographic interpretation, periodontal assessment, cephalometric analysis, plaque detection, and tooth identification, suggesting potential applicability to simulation-based learning and supervised clinical training, although educational effectiveness was not directly evaluated in all of these studies (60, 62–68, 79).

**Figure 4:**
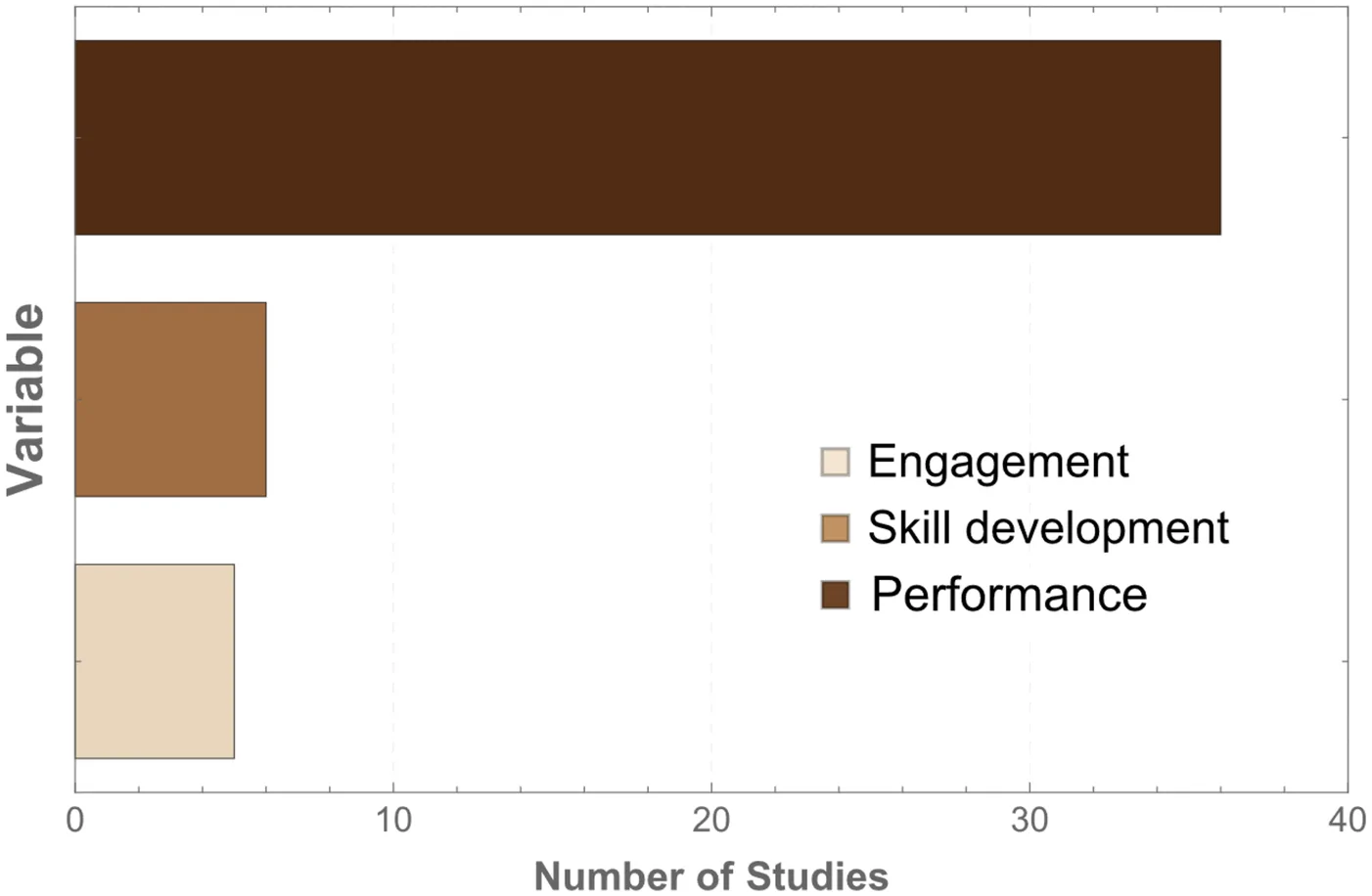
Distribution of included studies according to the variable of artificial intelligence implementation in dental education.

Although relatively few studies directly addressed skill development, some reported favorable findings for specific learning-related outcomes, including cephalometric landmark identification and individualized learning activities. These findings suggest that selected AI-assisted applications may support aspects of competency-based dental education, although the available evidence remains limited and heterogeneous. AI-supported cephalometric training was associated with greater landmark identification accuracy and lower inter-student variability in the evaluated studies (80, 81). Similarly, multimodal educational systems and RAG-enhanced learning environments were associated with favorable findings related to individualized learning, knowledge acquisition, and clinical reasoning (61, 64, 82). These findings suggest that selected AI applications may have potential to support aspects of competency-based dental education, although further direct educational evaluation is required.

Student engagement was the least frequently investigated educational variable despite its recognized importance in learning. Survey-based studies consistently reported favorable perceptions toward AI integration, with students perceiving potential benefits for learning efficiency, access to educational resources, and independent study (69–72, 83).

Figure 5 summarizes the direction of the findings reported across the included studies. Forty studies reported positive findings, four reported increased performance, and three reported negative findings. These results encompass both direct educational outcomes and clinical or technical outcomes with competency-linked educational relevance and therefore should not be interpreted uniformly as evidence of educational effectiveness. Nevertheless, the overall evidence suggests potential benefits of AI for selected aspects of dental education, particularly learner assessment, diagnostic reasoning, examination preparation, and selected competency-development activities. Positive outcomes were consistently observed in diagnostic accuracy, examination preparation, clinical decision-making, radiographic interpretation, personalized learning, and educational efficiency (49, 51–57, 60, 61, 73, 74, 77, 80, 81, 84). AI-assisted educational interventions also demonstrated the capacity to standardize instruction, provide immediate feedback, and reduce variability among learners, characteristics that are particularly valuable in competency-based dental education.

**Figure 5:**
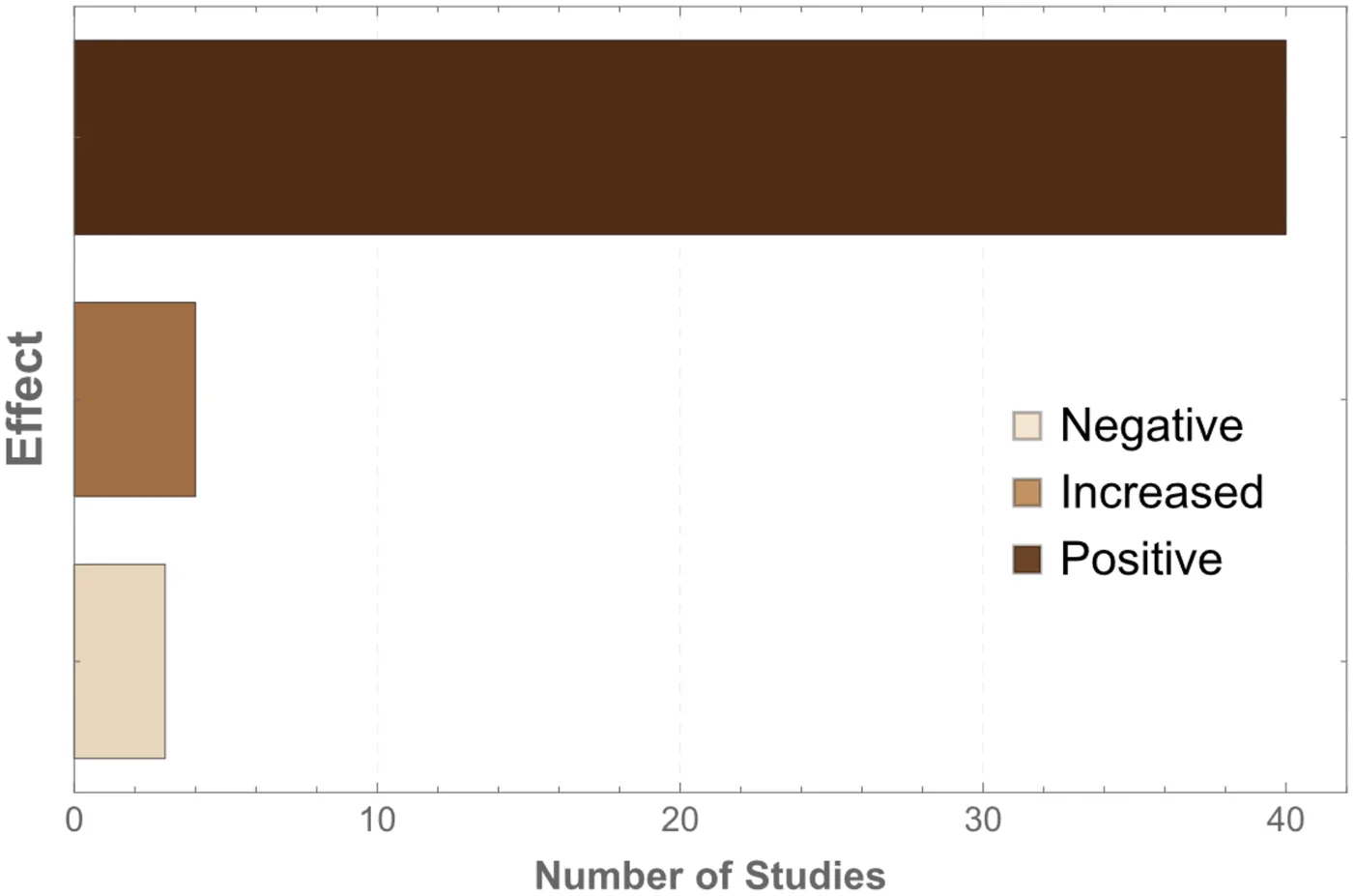
Overall educational effects reported in the included studies.

Studies categorized as reporting increased effects generally demonstrated that AI-enhanced educational approaches produced measurable improvements over conventional teaching strategies, particularly in orthodontic training, digital workflow education, and examination preparation (81, 85–87). These findings suggest that AI may function not only as a supplementary educational resource but also as a catalyst for improving educational efficiency and learner performance. Nevertheless, three investigations reported negative findings (88–90). These studies highlighted limitations including inconsistent responses generated by large language models, reduced diagnostic reliability in complex clinical scenarios, and discrepancies between AI-generated recommendations and evidence-based clinical guidelines.

### 3.2 Educational implementation and learning outcomes of artificial intelligence in dental education

As presented in Table 4, based LLMs, particularly ChatGPT and similar conversational AI systems, were the most frequently evaluated educational tools. These models were commonly employed to answer multiple-choice questions, explain clinical concepts, generate differential diagnoses, and provide feedback during self-directed learning activities (49–58, 75, 77, 78, 85, 87, 91). Across most studies, advanced LLM versions consistently demonstrated higher accuracy than earlier models, often achieving examination performances comparable to senior dental students while remaining inferior to experienced specialists in complex clinical reasoning tasks (49, 50, 54, 55, 77). These findings indicate that conversational AI may function effectively as a supplementary educational resource but should not replace faculty supervision or evidence-based instruction.

**Table 4:** Characteristics of artificial intelligence educational interventions in dental education, including delivery mode, learner activities, evaluation metrics, educational outcomes, and intervention duration.

Reference	Delivery Mode	Evaluation Metrics	Educational Outcomes
Pawar et al. (73)	Software-based analysis	AUC, accuracy, precision, recall, specificity	Models showed very high predictive performance; several algorithms reported AUC = 1.000 and accuracy/precision/recall/specificity >95%.
Durmazpinar, et al. (49)	Google Forms for students; ChatGPT-4o prompt-response testing	Correct answer rate, incorrect answer rate, unanswered rate, Kruskal–Wallis, Bonferroni *post hoc* tests, Fleiss' Kappa, Pearson Chi-square	ChatGPT-4o outperformed students: 91.4% correct versus 60.8% for 3rd-year and 79.5% for 5th-year students; differences were statistically significant.
Vazirizadeh; et al. (59)	Software/model-based image analysis	Accuracy, precision, recall, F1 score, ROC-AUC, confusion matrix, Grad-CAM visualization	ARN-CNN achieved 95% test accuracy and AUC 0.98, supporting automated detection of lip incompetence from orthodontic photographs.
Abou-Bakr; et al. (69)	Online	AI use by demographics/specialty; perceptions of advancement; concerns about reliability/originality; *p*-values	Students generally perceived AI positively; usage was moderate; younger participants and BDS holders reported higher AI use; need for AI education and responsible integration was identified.
León de la Fuente; et al. (85)	Online AI chatbot prompting	Reliability/accuracy, percentage agreement, Gwet's AC1, kappa statistics, Krippendorff's alpha, confidence intervals	ChatGPT-4o showed slightly higher reliability and greater repeatability than ChatGPT-4, but both models had only moderate accuracy and require expert supervision for educational use.
Çiçek; et al. (60)	Intraoral image-based digital analysis	IoU, Dice coefficient, recall, precision, t-tests, comparison with dentist segmentation	U-Net Transformer achieved IoU 0.7845 and outperformed three experienced pediatric dentists in plaque detection/segmentation, suggesting potential as a clinical support tool.
Yurt; et al. (50)	Asynchronous online questionnaire and chatbot prompting	Accuracy rate, correct/incorrect response ratio, ANOVA, Bonferroni *post hoc*, Chi-square, Cohen's kappa	Fifth-year students achieved highest accuracy (85.1%); ChatGPT-4o was comparable to fifth-year students and higher than third-year students and other chatbots.
Shukla; et al. (70)	Google Forms online questionnaire	Awareness percentages, attitudes toward AI, ethical awareness, year-of-study differences	Students showed generally positive attitudes toward AI and high belief in diagnostic benefits, but ethical awareness was low: only 26% were aware of ethical implications.
Fukuda; et al. (51)	Prompt-based interaction with ChatGPT-4o	Overall/domain accuracy; difference between text-only and visual questions	ChatGPT-4o showed reasonable proficiency, especially in basic knowledge areas, but weaker performance on visual, technical, orthodontic, pediatric, radiographic, and workflow-related items.
Sevimli; et al. (74)	In-person image interpretation with AI comparison	Diagnostic accuracy; *p*-values for differences between students and AI	AI showed better accuracy especially for caries and fillings and could improve undergraduate diagnostic accuracy for caries, fillings, and extractions.
Xu; et al. (61)	Online/chatbot-based multimodal interaction	5-point Likert scores: clinical accuracy, contextual relevance, completeness, professionalism/decision judgment, language fluency	EndoQ outperformed general LLMs with higher scores for accuracy (4.45±0.96), relevance (4.59±0.80), completeness (4.27±0.83), professionalism (4.45±1.06), and fluency (4.86±0.47).
Caponio; et al. (93)	Clinical patient-care setting	Implant survival rate; failure rate by student career level; hazard ratios for periodontitis and smoking	Overall implant survival was 95.1%; early-career students had higher failure rate (5.6%) than advanced students (3.7%), but experience was not independently significant after multivariate adjustment.
Suraiya, A.; et al. (62)	Offline computational model evaluation	Accuracy 0.8927; sensitivity 0.8327; specificity 0.8683; precision 0.8918; F1 score 0.8615; 92/100 bone-loss and 89/100 healthy cases identified	AI model showed satisfactory diagnostic performance and may be used as an adjunct to detect alveolar bone loss on panoramic radiographs.
Abdulmajeed. et al. (71)	Online self-administered questionnaire	Response rate 74%; familiarity 78.4%; AI-assisted diagnosis awareness 6.3%; AI clinical utilization 1.4%; high cost 54.5%; inadequate training 19.8%; limited availability 15.3%; specialists showed greater knowledge/utilization (*p* < 0.05)	Foundational digital tools were widely adopted, while advanced technologies including AI remained minimally integrated; study supports curriculum enhancement and continuing professional development.
Ji; et al. (52)	Text-based chatbot interaction	Open-ended scores for accuracy, completeness, structure, professionalism, and overall impression improved with CoT (p < 0.001); multiple-choice overall accuracy increased by 3.1%	Chain-of-Thought prompting improved ChatGPT-4 answer quality and accuracy, but authors caution it should not substitute for doctors.
Alnsour; et al. (94)	Text-based asynchronous chatbot Q&A	Usefulness, readability, quality, reliability, understandability, actionability, and inter-category differences	Most responses were useful/very useful (78.1%), generally reliable and understandable; however, readability was often difficult and actionability was limited.
Bostanci; et al. (63)	Offline model training and testing	Accuracy, precision, recall, F1-score, ROC-AUC	Accuracy and AUC were 0.80; macro precision/recall/F1 about 0.80, suggesting feasible AI-assisted apical status detection.
Felemban; et al. (53)	Text-based prompts entered independently into each model	Percentage accuracy, model comparison, answer consistency, speed/time, difficulty/cognitive category performance	ChatGPT-4 Turbo achieved 90% accuracy, ChatGPT-4 achieved 85%, and ChatGPT-3.5 achieved 78%; unpaid model improved after ten months while paid versions remained consistent.
Ghasemian; et al. (72)	Survey administration	Agreement percentages, attitude distributions, chi-square associations with demographic/educational factors, Cronbach's alpha	Students were generally positive: 80.4% agreed AI would advance dentistry, 75.7% valued radiographic analysis, 78.7% valued periodontal detection; caution remained about replacing dentists.
Ghorbani; et al. (64)	Asynchronous software-based image analysis	Sensitivity, precision, F1-score; overall sensitivity 99.89%, precision 95.72%, F1-score 97.76%	High diagnostic performance for automated tooth detection and numbering; weaker results for underrepresented primary incisors and third molars
Kandemir; et al. (75)	Text-based prompts and model-generated answers	Accuracy percentage; coverage score 1–5; inter-rater reliability *κ*=0.88; Cochran's Q, McNemar, Wilcoxon tests	ChatGPT-4 achieved highest accuracy (73.3%); DeepSeek-R1 63.3%, Gemini 55.0%, Claude 36.7%; stronger performance on knowledge than clinical questions
Eraslan; et al. (76)	Text-based prompts submitted to chatbots within a 7-day period	Accuracy rates; chi-square and z-tests; significance level 0.05; Copilot 73%, Gemini 63.5%, ChatGPT-3.5 61.1%, Claude Pro 57.9%, Perplexity 54.8%	Copilot performed best and significantly outperformed Perplexity; AI chatbots show promise as supplementary tools but are not reliable across all prosthodontics areas
Çeki˙ç; et al. (54)	Text-based prompts in Turkish submitted to LLM interfaces	Overall accuracy; MCQ accuracy; C-MCQ accuracy; *p*-values; model ranking	ChatGPT-4o achieved the highest overall accuracy (81.5%); Gemini 1.5 Flash was lowest (57%); C-MCQ performance was significantly lower than standard MCQs. LLMs were promising but not reliable as standalone sources.
Sabri; et al. (55)	Text-based question-answering through LLM platforms	Accuracy percentage; yearly scores; section performance; comparison with resident year groups	GPT-4 achieved the strongest performance (average 79.57%) and outperformed resident groups and other LLMs; Gemini exceeded early-year resident averages, while GPT-3.5 was weaker.
Ehrensperger; et al. (88)	Intraoral camera images processed by AI-based application	Cohen's kappa; agreement with BOP; agreement with dentist visual diagnosis; statistical significance	The AI application showed low agreement with BOP-based clinical diagnosis (kappa 0.055) and was not reliable for detecting gingivitis in its current form.
Sabbagh; et al. (80)	Computer-based self-directed learning	Angular/linear measurement deviations, performance variability, agreement percentages for usefulness, learning success	Students reported high acceptance (84.9% helpful, 86.5% greater learning success, 94.2% favored implementation); performance became more uniform, although most differences were not statistically significant.
Lin; et al. (81)	Computer-based supervised and self-directed digital training	Landmarking total scores before/after training; accuracy of skeletal, dental, and soft-tissue measurements	Digital training significantly improved landmarking accuracy; AI-assisted cephalometry helped minimize differences among students with different levels of clinical experience.
Erkal; et al. (89)	Prompt-based multimodal interaction with radiographs and clinical vignettes	Accuracy percentage and 95% CI by model/question; differences from expert	All LLMs scored significantly below the expert (expert 100%); models showed competence in basic tasks but failed to replicate expert-level reasoning for complex dens invaginatus classification and management.
Kaygısız,; et al. (65)	Asynchronous software-based image analysis	mAP, precision, recall, F1 score, and class-wise mean accuracy	YOLO v11 achieved high diagnostic performance: multi-class mAP 86%, precision 84%, recall 82%, F1 83%; class-wise accuracy 91% DC, 85% OKC, 82% RC.
Al-Jewari; et al. (95)	Face-to-face clinical care and follow-up	Dry socket incidence by sex, age, smoking, anesthetic dose, contraceptive use.	Dry socket was more common in the third decade of life and associated with female sex, higher anesthetic dose, contraceptive use, and smoking.
Zheng; et al. (86)	Software-based automated 3D reconstruction	Chamfer distance, visual geometric fidelity, processing time, and performance across 5%, 10%, 15%, and 20% simulated data loss	The model restored missing IOS data accurately, maintained average Chamfer distance below 0.01 in most conditions, and completed each point cloud in about 0.5 s.
Lafourcade; et al. (56)	Asynchronous API/chatbot interaction	Accuracy, repeatability, robustness, ability to identify chapters/categories and define terms	ChatGPT-4 and Claude-3 performed better than Mistral 7B and ChatGPT-3.5; course chapters sometimes improved accuracy, but robustness and consistency remained imperfect.
Keskin; et al. (84)	In-person image interpretation plus digital AI application	Kappa values, sensitivity, specificity, PPV, NPV, true/misdiagnosis counts	AI showed moderate agreement for caries and high agreement for dislodged restorations but weak agreement for periapical status; experts outperformed students and AI overall.
Simanoglu; et al. (77)	Asynchronous chatbot prompting	Correct response rate, total score, section scores, specialty performance	Both AI chatbots exceeded the passing threshold, with ChatGPT-4.0 outperforming Gemini Advanced, but both remained below top human performers and weaker in some specialties.
Kumaralingam; et al. (66)	Automated software-based analysis of intraoral ultrasound videos	Segmentation accuracy; mean bias and limits of agreement for ABL, GT, ABT; ICC values: ABL 0.893, GT 0.918, ABT 0.848	Accurate delineation of periodontal structures and consistent quantitative assessment; potential for real-time monitoring and personalized periodontal management
Marwaha; et al. (92)	Automated software pipeline applied to RVG images	Dice coefficient 0.89; accuracy 93.2%; sensitivity 91.4%; specificity 95.1%; F1-score 0.92; class-wise accuracy 95%, 92%, and 91%	Improved diagnostic consistency and efficiency; reduced diagnostic time and inter-observer variability
Büyüközer; et al. (90)	Text-based prompts in Turkish and English, with and without structured prompts	Inter-rater reliability 0.85–0.97; accuracy differences by model/language/prompt; no model reached ≥80% accuracy	English prompts improved accuracy for ChatGPT-4o; Claude performed best in selected conditions; overall accuracy insufficient for clinical reliance
Yu; et al. (82)	Automated software/model output from CBCT images	Dice similarity coefficient; 3D visualization; enamel thickness distribution	High segmentation accuracy reported (Dice score 96.6%) and clinically useful 3D enamel visualization.
Jalali; et al. (57)	Text-based chatbot prompts and generated explanations	Accuracy; consistency across 3 attempts; ordinal reasoning score 0–2; statistical comparisons	Accuracy ranged from 48% to 71%; several chatbots exceeded 70%, but results showed important limitations and need for supervision.
Tosun; et al. (78)	Text-based chatbot responses to multiple-choice questions	Overall accuracy; accuracy by chatbot; accuracy by case-based vs. knowledge-based questions; statistical significance	ChatGPT-4 achieved the highest accuracy (75.8%); models performed better on knowledge-based than case-based questions, supporting cautious educational use.
Slashcheva; et al. (83)	Live webinar, asynchronous webinar, clinical chairside use	Provider expectations/experience; perceived accuracy and efficiency; patient helpfulness; software utilization	Most providers viewed AI annotations as helpful for patient education; patients reported improved understanding and 92% intended to follow treatment recommendations; workflow impact was mixed.
Camlet; et al. (87)	Text prompt interaction with AI chatbot	Percent correct; pass threshold; odds of correct answer; *p*-values; difficulty index	ChatGPT-4 outperformed ChatGPT-3.5 and exceeded the 60% passing threshold in the English Spring 2023 exam; performance varied by language and difficulty.
Enkvetchakul; et al. (67)	Software/application-based AI diagnostic system	Accuracy on TD-1/TD-2; response time; SUS score; comparison with single models	Model achieved high classification accuracy (98.87% and 98.41%), rapid response time (1.5 s), and high usability score (SUS 94.5), suggesting strong diagnostic support potential.
Hong; et al. (79)	Digital/offline model evaluation	Accuracy, F1-score, mAP@50, Cohen's kappa	AI assistance improved junior dentists' agreement with experts and reduced evaluation time; ENPAT showed strong real-world grading performance.
Khurshid; et al. (68)	Offline digital model training/testing	Accuracy, precision, F1-score, mAP, IoU-based detection metrics	ViT achieved highest accuracy, precision and F1-score; Faster R-CNN showed strongest, supporting AI-assisted radiographic detection.
Çakmak; et al. (58)	Digital online chatbot interaction	Accuracy rate, performance by question type	Chatbots showed strong text-based reasoning but weak visual interpretation; Copilot had highest overall accuracy, while ChatGPT-5 provided more reliable academic sources.
İşisağ; et al. (91)	Text-based prompts in a new ChatGPT chat window	Accuracy rate; repeatability/reproducibility; response consistency	High accuracy for simple fixed prostheses (97.08%) but low accuracy for complex fixed prostheses (19.69%); overall accuracy 52.3%; high time-of-day concordance; AI useful as supplementary aid but not reliable alone for complex planning.

ABL, alveolar bone level; ABT, alveolar bone thickness; AI, artificial intelligence; AUC, area under the curve; BOP, bleeding on probing; CI, confidence interval; CoT, Chain-of-Thought; F1, F1 score; GT, gingival thickness; ICC, intraclass correlation coefficient; IoU, intersection over union; mAP, mean average precision; NPV, negative predictive value; PPV, positive predictive value; ROC-AUC, receiver operating characteristic area under the curve; RVG, radiovisiography; SUS, system usability scale; MCQ, multiple-choice question .

The evidence summarized in Table 4 also highlights the technical capabilities of AI-assisted image analysis in areas potentially relevant to dental education. Several investigations evaluated convolutional neural networks, transformer architectures, and automated segmentation algorithms for identifying caries, periodontal disease, cephalometric landmarks, lip incompetence, enamel morphology, cyst classification, and other oral conditions (59, 60, 62–68, 73, 79, 82, 84, 86, 88, 92). Many of these systems achieved high diagnostic performance, frequently reporting strong accuracy, AUC, Dice coefficient, or F1-score values. However, where these studies did not directly evaluate learners, educational interventions, or training outcomes, their findings are interpreted as evidence of potential applicability to simulation, supervised diagnostic training, radiographic interpretation practice, or competency development rather than as direct evidence of educational effectiveness. Their educational value therefore remains to be established through dedicated learner-centered studies. Nevertheless, some applications exhibited limited agreement with expert clinical diagnosis, particularly for complex periodontal assessments, emphasizing that AI-generated outputs require careful interpretation and instructor validation (84, 88).

Most participants believed that AI would positively influence dental education and future clinical practice while recognizing its value for improving diagnostic efficiency and facilitating access to educational resources. But, respondents also expressed concerns regarding ethical issues, reliability, academic integrity, and excessive dependence on automated recommendations (69–72). Particularly noteworthy was the relatively low awareness of ethical implications reported in some cohorts, suggesting that AI literacy should extend beyond technical competency to include responsible use, transparency, privacy, and professional accountability (70). Another important finding emerging from Table 4 is the wide variation in educational delivery methods. Learning activities ranged from asynchronous chatbot interactions and online questionnaires to supervised computer-assisted radiographic training, image annotation exercises, clinical simulations, and face-to-face patient management supported by AI systems (49, 50, 69, 74, 80, 81, 83).

### 3.3 Applications, competency development, and challenges of artificial intelligence in dental education

Table 5, show diagnostic education remains the principal area in which AI has been incorporated into undergraduate and postgraduate dental training. Most investigations evaluated AI systems designed to assist learners in interpreting radiographs, recognizing oral diseases, identifying anatomical landmarks, or predicting treatment outcomes (59, 60, 62, 64–68, 73, 79, 82, 84, 88, 92). These educational strategies commonly relied on deep learning algorithms, convolutional neural networks, transformer architectures, or automated image segmentation models that provided immediate feedback on learner performance. The consistently high diagnostic accuracy reported in many studies suggests that AI has considerable potential to improve students' visual interpretation skills while reducing variability in clinical assessments (59, 60, 65, 67, 73, 92). Nevertheless, several investigations also demonstrated that AI performance declined in complex diagnostic scenarios requiring contextual clinical reasoning, reinforcing that these technologies should complement rather than replace instructor-guided education (79, 84, 88).

**Table 5:** Characteristics of studies evaluating artificial intelligence in dental education according to dentistry field, study design, dental competency domain, curriculum area, educational strategies, assessment methods, reported barriers, and study limitations.

Reference	Dentistry Field	Dental Competency Domain	Educational Strategies	Assessment Method	Reported Barriers	Limitations
Pawar et al. (73)	Periodontics	Diagnosis, risk assessment, treatment planning	Data-driven predictive modelling	Algorithm performance testing against prognosis/tooth-loss outcomes	Small sample size; retrospective data bias; limited external validation and need for larger/diverse datasets.	Small sample size; retrospective design; potential selection bias; requires external validation on larger and more diverse populations before clinical implementation.
Durmazpinar, et al. (49)	Endodontics	Diagnosis, clinical reasoning, radiographic interpretation	Case-based assessment using clinical images.	Multiple-choice diagnostic test with response categories correct/incorrect/unanswered	Constrained by one student population, narrow clinical cases, MCQ format, lack of open-ended assessment	Limited endodontic case variety; assessed only multiple-choice diagnostic tasks; single student population; variability in student experience.
Vazirizadeh; et al. (59)	Orthodontics	Diagnosis, image interpretation, orthodontic assessment	Deep-learning model training with preprocessing.	Model validation on test set and cross-validation	Needs larger and more diverse datasets, validation in malocclusion/craniofacial variation cases	Performance may be affected by image quality, facial positioning, and cropping errors; limited dataset; requires validation with larger and more diverse populations.
Abou-Bakr; et al. (69)	General dentistry/multiple specialties	Professionalism, digital literacy, evidence-based practice	Self-administered questionnaire.	Self-reported questionnaire responses	Concerns about AI accuracy, ethical implications, originality in research, integration challenges, and lack of structured AI training	Cross-sectional self-report design; sample restricted to Egyptian postgraduate students
León de la Fuente; et al. (85)	Endodontics	Clinical reasoning, diagnostic/therapeutic knowledge, evidence-based practice.	Question-based prompting.	Expert 3-point Likert scoring: incorrect, partially correct/incomplete, correct	Moderate reliability; inconsistent or partially correct answers; risk of omissions, bias.	Only closed clinical questions in endodontics were tested; no direct student learning intervention.
Çiçek; et al. (60)	Pediatric dentistry/preventive dentistry	Diagnostic skill, preventive assessment, clinical decision support	Dataset collection, annotation, model training, testing.	Algorithmic performance metrics and comparison with expert dentists	Small dataset; single camera type; lack of public plaque dataset for external comparison	Relatively small image dataset despite augmentation; uncertain generalizability to other devices; model predicts plaque presence/segmentation but not density; limited external validation.
Yurt; et al. (50)	Endodontics	Diagnostic reasoning and clinical decision-making	Case-based multiple-choice assessment aligned with clinical guidelines	15 closed-ended multiple-choice questions; responses independently reviewed by two researchers.	LLM response variability; limited generalizability to text-based MCQs; differences in clinical experience	Single-center design; limited to endodontic pulpal/periapical scenarios; text-based multiple-choice format does not directly measure full clinical competence
Shukla; et al. (70)	General dentistry/Dental education	Professionalism, ethics, digital literacy, and clinical decision awareness	Self-reported knowledge, attitude, and perception.	Structured questionnaire; descriptive statistics and Chi-square test.	Low ethical awareness; uncertainty about AI implications.	No intervention or objective skill/performance testing; self-reported survey data; cross-sectional design limits causal inference; possible selection and response bias.
Fukuda; et al. (51)	Dental technology/Prosthodontic laboratory education	Knowledge application, examination reasoning, and technical dental technology competence	Standardized prompting.	Japanese National Dental Technician Examination questions; Fisher's exact test.	Lower accuracy for visual elements, radiographs, procedural workflows	Single AI model evaluated; limited to Japanese dental technician exam questions; visual and technical content remained challenging.
Sevimli; et al. (74)	Dentomaxillofacial radiology/diagnostic dentistry	Diagnosis and treatment planning	Benchmarking learner diagnoses against AI output	Comparison with reference/AI diagnostic results; chi-square analysis	AI sensitivity varies by modality; need for large data; possible misuse/interpretation errors.	Panoramic radiography has diagnostic limitations; AI disadvantages include dependence on data quality/modality and multidisciplinary implementation challenges.
Xu; et al. (61)	Endodontics	Clinical reasoning and knowledge application	Personalized question answering; evidence-grounded retrieval.	Expert blinded rating against general-purpose LLMs	Generic LLMs may lack clinical accuracy, contextual grounding, and pedagogical clarity.	Evaluation used a limited expert-validated question bank and expert ratings rather than direct student learning outcomes; implementation with real learners and longitudinal outcomes remains to be tested.
Caponio; et al. (93)	Implant dentistry/periodontology	Clinical procedural competence and patient-outcome management	Supervised clinical practice and retrospective outcome evaluation	Implant survival/failure analysis	Patient risk factors such as periodontitis and smoking increased failure risk; case allocation to advanced students may bias experience comparisons	Retrospective design; some influential factors not measured; follow-up was medium-term only; patient adherence and oral hygiene engagement were not assessed; no direct AI educational intervention was tested.
Suraiya, A.; et al. (62)	Periodontology/Dental radiology	Diagnostic competence; radiographic interpretation	Supervised image classification using expert-labeled radiographs	Confusion matrix; comparison with expert-labeled cases	Need for clinical periodontal evaluation; image quality and 2D panoramic limitations; need for training on CNN use and patient-history integration	Limited validated image sample; low panoramic image resolution; overlapping structures; 2D imaging limits complete diagnosis; CNN alone cannot establish definitive periodontal diagnosis.
Abdulmajeed. et al. (71)	Endodontics/Dental education	Technology literacy; clinical decision-making; evidence-based endodontic practice	Needs assessment; comparison by professional category	Validated 22-item questionnaire; descriptive statistics; Pearson chi-square test	High equipment cost; inadequate training; limited availability; limited undergraduate training; infrastructural disparities	Cross-sectional self-reported survey; geographically limited; possible response and selection bias; no objective measure of clinical competence or patient outcomes.
Ji; et al. (52)	Oral and maxillofacial surgery/Dental public education	Knowledge acquisition; clinical reasoning support; communication of oral-health information	Chain-of-Thought prompting to structure reasoning.	Expert rating of open-ended answers; multiple-choice accuracy comparison; Mann–Whitney U test	Not a substitute for physicians/dentists; risk of relying on AI answers without professional judgment	Question set and evaluation context may not represent all clinical scenarios; limited to ChatGPT-4 and selected prompts; AI responses require expert oversight; public-use implications need caution.
Alnsour; et al. (94)	Oral health/dental public health; smoking-related dental health information	Patient education, communication, prevention, and health promotion	Prompting individual questions; use of validated scoring rubrics.	Expert reviewer rating using usefulness scale, FKG, SMOG, GQS, CLEAR, and PEMAT; statistical tests included ANOVA	Specialized topics were less reliable/useful; many responses were hard to read; actionability was low.	No layperson evaluation; only text-based responses assessed; findings may not generalize to multimodal AI formats or broader non-expert audiences
Bostanci; et al. (63)	Endodontics; pediatric dentistry; oral and maxillofacial radiology	Diagnostic reasoning; radiographic interpretation; clinical decision-making	Supervised learning using labeled images; comparison with reference labels	Model performance evaluation	Need for sufficient labeled CBCT-derived images; limited generalizability to other populations or imaging protocols	Small dataset; retrospective single-context imaging data; model requires external validation before educational or clinical adoption
Felemban; et al. (53)	Oral and maxillofacial pathology; oral radiology	Knowledge recall; diagnostic reasoning; interpretation of oral pathology and radiology concepts	Prompting MCQs; expert-validated item difficulty	Correct/incorrect scoring against expert-validated answer key	Limitations in complex cognitive questions and lack of image-based question handling; potential inconsistency across model versions	MCQs were mainly knowledge-based and text-only; findings may not generalize to image-rich diagnostics or higher-order clinical reasoning
Ghasemian; et al. (72)	General dentistry; dental education; AI in clinical dentistry	Professionalism; digital competence; evidence-informed clinical decision-making	Stratified sampling; validated attitude questionnaire; descriptive and chi-square analysis	Self-reported questionnaire	Knowledge gaps, ethical concerns, professional autonomy concerns, skepticism about AI replacing dentists	Single-country context; self-reported attitudes; cross-sectional design cannot establish change over time or actual AI-use competency
Ghorbani; et al. (64)	General dentistry; pediatric dentistry; diagnostic imaging; teledentistry	Diagnosis and treatment planning; radiographic/photographic interpretation; digital dentistry	Faculty-guided annotation, supervised dataset labeling.	Automated model testing against annotated reference labels	Class imbalance and underrepresentation of primary incisors/third molars; access to younger patients; misclassification in uncommon tooth groups	Retrospective dataset; unequal representation of tooth classes; lower precision for some primary teeth and third molars; external validation needed
Kandemir; et al. (75)	Periodontology	Periodontal knowledge; clinical reasoning; evidence-based decision support	Question categorization, standardized prompting.	Multiple-choice accuracy scoring and coverage rubric	Lower accuracy on clinical decision-making questions; variable performance between models; need for expert verification	Only 60 public periodontology questions; limited generalizability to other dental disciplines and exam formats; publicly available questions may not represent all curriculum content; LLM outputs may vary over time
Eraslan; et al. (76)	Prosthodontics	Prosthodontic knowledge; clinical reasoning; materials and treatment planning support	Subtopic grouping, standardized question presentation.	Correct/incorrect scoring of multiple-choice answers	Uneven chatbot accuracy by model and topic; lower performance in removable partial dentures; language translation may affect responses.	Questions translated into English; only one exam source and 126 questions; results may change as models update; chatbots are not consistently reliable clinical/educational tools
Çeki˙ç; et al. (54)	Endodontics	Knowledge application, diagnosis, clinical reasoning, treatment planning	Benchmark testing; comparative model evaluation	Automated comparison with official answer keys; statistical analysis in SPSS	Variable accuracy by model and question type; weaker performance on combination-type MCQs; risk of overreliance	Restricted to publicly available Turkish endodontic exam questions; no real student intervention; outputs may change across model updates; not a direct clinical competence assessment
Sabri; et al. (55)	Periodontology	Knowledge, evidence-based reasoning, clinical decision-making	Comparative testing; benchmarking against resident cohorts.	Answer accuracy compared with examination key and resident scores; chi-square statistical testing	Uneven model performance; lower reliability of GPT-3.5 and Gemini compared with GPT-4	Limited to standardized exam questions; no direct teaching intervention; model versions may change; results may not generalize to clinical skill performance
Ehrensperger; et al. (88)	Periodontology/preventive dentistry	Diagnosis, screening, prevention, patient communication	Visual screening comparison; reference-standard comparison	Comparison with clinical diagnosis based on bleeding on probing and dentist photo-based visual assessment	Gingivitis is difficult to detect visually; low agreement with clinical gold standard; current AI filter lacks reliability	Small, young, convenience sample; limited gingival diversity; single examiner; image-quality and algorithm-training constraints.
Sabbagh; et al. (80)	Orthodontics	Radiographic interpretation; cephalometric landmark identification; orthodontic diagnostic competence	Immediate feedback; repetitive practice; comparison with AI/reference landmarks.	Comparison of student tracing measurements with expert/reference standards and post-use acceptance questionnaire	Need for AI literacy; unresolved reference-standard issues; possible mismatch between digital practice and manual assessment.	Single-institution study; effectiveness may be underestimated because training was digital but assessment was manual; expert consensus reference standards may contain bias; residual AI landmark errors; amount of student practice time was not assessed.
Lin; et al. (81)	Orthodontics	Cephalometric landmark identification; radiographic analysis; orthodontic diagnostic competence	Real-time feedback; visual error correction; paired student workintroductory teaching	Pre- and post-training score comparison; comparison of AI-assisted measurement accuracy among students from different grades	Traditional teaching workload, limited teaching time, delayed individual feedback, and need for digital competence.	Limited sample size; AI-assisted task involved verifying/adjusting AI landmarks rather than fully independent landmarking; findings should be interpreted cautiously and may not generalize to all settings.
Erkal; et al. (89)	Endodontics	Clinical reasoning; radiographic diagnosis; anomaly classification; treatment planning and prognosis in endodontics	Case-based questioning; expert benchmarking; structured diagnostic reasoning prompts	Responses scored against reference key; accuracy compared using Kruskal–Wallis, Mann–Whitney U, and Chi-square tests	LLMs struggled with complex anomaly classification and visual clinical reasoning; Gemini and DeepSeek were excluded because image upload/visual input was unsupported in this task.	Small case set (*n*=19); radiograph quality heterogeneity; model outputs may vary over time; results may not generalize to other endodontic conditions, prompts, or model versions.
Kaygısız; et al. (65)	Oral and maxillofacial radiology/oral and maxillofacial surgery	Diagnostic reasoning and radiographic interpretation	Model-based decision support; performance benchmarking	Algorithmic performance assessment against annotated reference labels	Small and imbalanced dataset; similar radiographic appearance of cyst types; need larger external validation.	Further validation on larger balanced datasets recommended; should not replace expert diagnosis; possible limited generalizability.
Al-Jewari; et al. (95)	Oral and maxillofacial surgery	Clinical decision-making and patient management	Case-based clinical observation; postoperative instruction	Clinical assessment at follow-up visits	Patient risk factors such as smoking, contraceptive use, high anesthetic dose, and extraction difficulty; no AI component.	Single teaching-hospital sample; small sample size; verbal consent; limited control of confounders; no AI/educational technology intervention.
Zheng; et al. (86)	Digital dentistry/orthodontics/intraoral scanning	Digital workflow competence and technical accuracy	Simulation-based learning; digital workflow optimization	Algorithmic reconstruction assessment against ground-truth point clouds	Incomplete IOS data from complex intraoral environments; scanner variability; need integration into real-world workflows.	Subtle differences remained in distribution and edge morphology; no comparison with other state-of-the-art completion methods; scanner-brand/settings validation still needed.
Lafourcade; et al. (56)	Restorative dentistry and endodontics	Knowledge, diagnosis/reasoning, evidence-based decision support	Question-answering, prompt engineering, comparative benchmarking.	Expert answer key comparison; repeated and paraphrased prompts; statistical comparison across models	Inconsistent robustness; hallucination risk; dependence on prompt wording; limited context windows; need for validated domain-specific material	Moderate performance despite advanced models; variable accuracy across closely related concepts; inconsistency when course chapters were supplied; benchmark did not measure real learner outcomes directly
Keskin; et al. (84)	Endodontics; oral and maxillofacial radiology; restorative diagnosis	Clinical diagnosis, radiographic interpretation, treatment follow-up assessment	Case-based radiographic assessment, expert benchmarking	Gold-standard comparison with endodontist conclusions; kappa agreement; sensitivity/specificity analysis	Lower AI specificity/PPV for periapical status; panoramic-image distortion; anatomical superimposition	Retrospective single-center sample; panoramic radiographs have lower resolution than intraoral images; limited sample after inclusion criteria; AI performance varied by diagnostic category
Simanoglu; et al. (77)	General dentistry; dental specialty examination; dental education	Knowledge acquisition, examination performance, clinical and basic-science reasoning	Standardized test simulation, comparative scoring, human-AI benchmarking	Official answer key comparison; Pearson chi-square statistical testing	LLMs not designed specifically for dentistry;possible differences in access to online data/training cutoff; weaker basic/clinical subsection performance	Only two exam years; excluded post-2021 questions; evaluated answer accuracy rather than real educational impact; results may vary with model updates and prompting
Kumaralingam; et al. (66)	Periodontics/dental imaging	Patient assessment, diagnosis, imaging interpretation, treatment planning	Expert annotation, algorithm training/validation,	Comparison with expert-annotated ground truth; Bland–Altman agreement; intraclass correlation coefficients	Manual assessment is time-consuming; inter-operator variability; static image limitations; need for real-time assessment	Single-center dataset; limited sample size of patients; education outcomes not assessed; clinical deployment/generalizability require further validation
Marwaha; et al. (92)	Conservative dentistry, endodontics, dental radiology	Diagnosis, radiographic interpretation, clinical decision making	Expert annotation, supervised model training, validation, and testing	Expert annotation comparison; held-out test-set evaluation	Manual RVG interpretation is subjective, variable, and time-consuming; early-stage lesions may be missed	Retrospective data; possible need for larger multicenter validation; no direct educational outcomes or learner performance measures reported
Büyüközer; et al. (90)	Endodontics/regenerative endodontics	Evidence-based decision making, guideline knowledge, clinical reasoning	Prompting, multilingual query design, expert rubric-based evaluation	Two-specialist assessment using a four-point rubric; statistical analysis of accuracy and reliability	Language variability; prompt sensitivity; AI hallucination/inaccuracy risk; model performance below clinical reliability threshold	Limited to selected questions and models; AI systems may change over time; findings may not generalize beyond Turkish/English or REP topics
Yu; et al. (82)	Dental radiology; restorative dentistry; endodontic/orthodontic treatment planning	Diagnosis and treatment planning; digital imaging; patient care	Demonstration of AI-generated 3D enamel models and quantitative thickness maps	Segmentation performance compared with manual annotation	Need for annotated CBCT data; clinical deployment and chair-side integration still require validation	Small-sample model development; retrospective design; education outcomes not directly measured
Jalali; et al. (57)	Endodontics	Knowledge application; clinical reasoning; evidence-based decision-making	Board-style MCQ practice; immediate answer selection	Expert-scored correctness and reasoning quality	Hallucinations, inconsistent responses, incorrect rationales, lower performance on some literature-based/specialized content	Questions were board-style/expert-generated rather than an official ABE exam; text-only MCQs; findings may change as models update
Tosun; et al. (78)	Prosthodontics	Knowledge recall; clinical reasoning; problem solving	MCQ-based exam practice; comparison of AI-generated answers	Correct-answer scoring against DUS answer key	Inaccurate answers, weaker case-based reasoning, misinformation risk, ethical concerns, need for evidence-based validation	Limited to prosthodontics MCQs from one national exam; only certain chatbot versions; no direct student learning outcomes measured
Slashcheva; et al. (83)	Radiology; cariology; periodontology; clinical dental care	Diagnostic reasoning; patient communication; treatment planning	Use of AI visual aids during patient explanation	Provider surveys before/after implementation; patient feedback survey; utilization reports	Workflow burden for hygienists/assistants; time needed to open software for each patient; role-specific relevance	Small patient sample; unclear provider inclusion/exclusion criteria; no detailed provider demographics; login-based utilization may not identify actual user; short follow-up
Camlet; et al. (87)	Periodontology	Knowledge application; clinical decision making; evidence-based periodontology	Question answering, benchmarking	Multiple-choice specialty examination performance	Incorrect answers on harder questions; language-dependent performance; ethical concerns about verification and AI-generated content	Only two exam sessions; single-best-answer format; manual translation; AI model versions/time-bound behavior
Enkvetchakul; et al. (67)	Oral radiology; endodontics; periodontology; dental diagnostics	Radiographic diagnostic skill; disease classification; clinical decision support	Model training, validation, decision fusion	Model accuracy testing; response-time measurement; System Usability Scale feedback	Need for broader external validation; complexity of ensemble/metaheuristic methods	Proprietary datasets limit reproducibility/generalizability; educational learners not directly studied; further multi-center validation needed
Hong; et al. (79)	Periodontology/Orthodontics	Diagnostic reasoning and periodontal assessment	AI-assisted interpretation	Expert-reference performance comparison	Need image labelability and generalization across broader clinical settings; performance varies by grading task	Retrospective dataset; limited real-world external validation; focused on anterior esthetic zone and image-based indices
Khurshid; et al. (68)	Dental radiology/Prosthodontics	Radiographic interpretation and prosthodontic/restoration identification	Supervised model training; model benchmarking	Model performance testing	Need clinician-in-the-loop validation, independent multicenter testing, and workflow integration.	Model requires broader external validation; limitations include dataset dependency and need for structured user studies
Çakmak; et al. (58)	Orthodontics	Orthodontic knowledge, reasoning and source evaluation	Question answering; Bloom's taxonomy categorization	Answer accuracy and citation/source assessment	Visual-analysis weakness; variable citation quality; risk of unreliable sources	Limited to 129 orthodontic DUS questions; performance may change as chatbot versions update; visual questions had lower reliability
İşisağ; et al. (91)	Prosthodontics/fixed prosthodontics	Treatment planning; prosthodontic clinical reasoning; abutment selection	Case-based questioning; repeated prompting	Expert evaluation coded as correct/incorrect; statistical analysis of accuracy and agreement	Low accuracy in complex prosthodontic cases; possible misleading or incorrect outputs; limited contextual understanding	Only textbook-based idealized scenarios were used; no real patients; no caries, periodontal, esthetic, tooth-position, radiographic, intraoral scan, photo, video, or broader clinical variables; cantilever and long-span posterior scenarios excluded.

Another prominent finding illustrated is the rapid adoption of LLMs as educational tools. Numerous studies assessed conversational AI systems using case-based discussions, multiple-choice examinations, short-answer questions, and clinical problem-solving exercises (49–58, 75–78, 85, 87, 91). Overall, newer generations of LLMs consistently outperformed previous versions and frequently achieved examination scores comparable with senior dental students, particularly in knowledge-based questions (50, 53–55, 77). However, performance decreased when questions required advanced clinical judgment, interpretation of complex radiographic findings, or multidisciplinary treatment planning (51, 78, 89, 91). These findings support the role of LLMs as supplementary educational resources capable of enhancing self-directed learning, while emphasizing the continued necessity of expert supervision and critical appraisal.

Numerous studies reported applications targeting professionalism, evidence-based practice, digital literacy, communication, and treatment planning (69, 71, 72, 83). Questionnaire-based investigations consistently showed positive student attitudes toward AI integration, with most participants recognizing its potential to improve learning efficiency, diagnostic confidence, and clinical productivity (69–72). Despite this optimism, concerns regarding reliability, ethical implications, data privacy, academic integrity, and excessive dependence on AI-generated recommendations were frequently reported (69–72). Learning activities included supervised image analysis, chatbot-assisted tutoring, self-directed online learning, case-based simulations, questionnaire-based instruction, automated feedback systems, and AI-supported clinical training (49, 50, 69, 74, 80, 81, 83). Such diversity reflects the flexibility of AI technologies to support multiple instructional approaches across both preclinical and clinical education. Importantly, studies evaluating supervised AI-assisted training reported improvements in diagnostic consistency, landmark identification, radiographic interpretation, and learner confidence following repeated interaction with AI-generated feedback (74, 79–81). These findings align with competency-based education models that emphasize deliberate practice, continuous feedback, and individualized learning pathways.

Table 5 highlights several methodological barriers that limit interpretation of the current evidence. Frequently reported challenges included small sample sizes, retrospective study designs, limited external validation, single-center recruitment, restricted clinical datasets, and variability in AI algorithms (49, 59, 62, 65, 67, 69, 73, 84, 88). Additionally, many investigations evaluated technical performance rather than educational effectiveness, making it difficult to determine whether improvements in AI accuracy translate into enhanced long-term clinical competence. The diversity of assessment methods—including algorithmic accuracy metrics, multiple-choice examinations, perception surveys, Likert scales, and expert agreement analyses—also complicates direct comparison across studies (49–95). This methodological heterogeneity underscores the need for standardized educational outcome measures and validated competency frameworks for future research. Another important observation emerging is that reported study limitations were remarkably consistent regardless of specialty. Studies frequently emphasized the need for larger multicenter datasets, prospective validation, more diverse patient populations, and integration of AI into authentic educational environments before widespread curricular implementation (59, 62, 65, 69, 73, 79, 84, 88).

## 4 Discussion

### 4.1 Current landscape and evolution of artificial intelligence in dental education

These findings reflect growing interest in exploring AI as a potential complementary educational resource rather than solely as a diagnostic or technological tool (49–56, 59–66, 69–77, 80, 81, 84–86, 88, 89, 93–95). Early applications mainly involved machine learning, deep learning, and convolutional neural networks designed to improve diagnosis through automated image interpretation and predictive analytics. These models were used to detect oral diseases, classify radiographic findings, predict tooth prognosis, identify orthodontic landmarks, assess alveolar bone loss, and segment dental plaque (59, 60, 62–65, 73, 84, 86). Although initially developed for clinical decision support, these systems may have educational applicability as reference tools for supervised diagnostic interpretation, simulation, and competency-based practice. However, this potential should be distinguished from demonstrated educational effectiveness unless learner outcomes or training interventions were directly evaluated.

More recently, large language models and generative platforms such as ChatGPT, GPT-4o, Gemini, Microsoft Copilot, Claude, DeepSeek, and RAG-based assistants have expanded AI applications beyond diagnosis. These tools have been evaluated or proposed for applications including interactive tutoring, personalized feedback, examination preparation, and real-time clinical consultation (49–56, 61, 75–77, 85, 89). Their conversational capabilities may offer opportunities for explanation, case exploration, self-directed learning, and competency-based educational activities. AI use has also diversified across dental specialties. Endodontics was the most frequently studied area (49, 50, 54, 61, 85), but applications were reported in orthodontics, periodontology, pediatric dentistry, prosthodontics, implant dentistry, oral radiology, oral pathology, and dental technology education (51, 55, 59, 60, 62, 64, 65, 80, 81, 84). Studies from Asia, Europe, the Middle East, and Australia demonstrate international adoption (49–56, 59–66, 69–77, 80, 81, 84–86, 88, 89, 93–95). Most reported favorable perceptions, particularly regarding diagnostic accuracy, learning efficiency, rapid feedback, and independent learning (69, 70, 72). Much of the evidence comes from cross-sectional studies, pilot interventions, and performance comparisons rather than curricular integration (49, 50, 72, 80, 81). Although some models matched or surpassed students in examinations and diagnostic tasks, performance was less reliable in complex reasoning, multimodal interpretation, and decisions (51, 52, 54, 56, 85, 89).

### 4.2 Impact of artificial intelligence on learning outcomes and student performance

Across specialties and educational settings, AI-assisted tools improve knowledge acquisition, diagnostic accuracy, clinical reasoning, technical skills, and self-directed learning, supporting competency-based education (49–56, 59–66, 69–77, 80, 81, 84–86, 88, 89, 93–95). Diagnostic performance is among the most consistently reported benefits. In endodontics, ChatGPT-4o outperformed third- and fifth-year students in case-based questions and showed greater consistency in clinical decision-making (49). Other comparisons found that ChatGPT-4o performed similarly to senior dental students and surpassed ChatGPT-3.5, Gemini, and Microsoft Copilot in guideline-based scenarios (50). LLMs also achieved passing scores in national and specialty examinations, demonstrating value for knowledge reinforcement and examination preparation (51, 54, 55, 75–77). But, performance declined in complex radiographic, procedural, and visually demanding tasks (51, 54, 56). These effects should be interpreted according to study context: improvements measured directly in learners represent educational evidence, whereas diagnostic or technical performance reported without a learner-centered evaluation indicates competency-linked potential rather than demonstrated educational effectiveness.

AI also supports clinical reasoning and practical training. RAG-based systems generated more accurate and contextually relevant responses than general-purpose models (61), while chain-of-thought prompting improved response organization and clinical accuracy (52). In orthodontics, AI-assisted cephalometric training improved landmark identification (80, 81), and AI-supported radiographic interpretation enhanced detection of caries, restorations, and extractions (74). Students generally valued AI for rapid information retrieval, personalized feedback, and independent study (69, 70, 72). Nevertheless, concerns remain regarding overreliance, ethical awareness, and inaccurate or incomplete responses in complex cases (52, 56, 85, 89). Therefore, AI should remain a complementary resource under faculty supervision. Curricula should combine AI-supported learning with critical appraisal, professionalism, ethical reasoning, and responsible technology use to preserve sound clinical judgment and lifelong learning. This approach ensures students critically verify outputs against scientific evidence independently.

### 4.3 Comparison with previous reviews and current evidence

The studies summarized in Table 6 are secondary reviews used for contextual comparison with the present systematic review and do not constitute part of the primary evidence synthesis.

**Table 6:** Comparison of previous reviews on artificial intelligence in dentistry and dental education with the present systematic review.

Author	Focus Odontology Area	Tools Reported	Barriers	Employed
Assiry al. (96)	Pediatric Dentistry and Special Care Dentistry	AI, ML, DL, Convolutional Neural Networks (CNN), NLP, AI chatbots, teledentistry, CBCT, virtual reality (VR), robotics	Limited pediatric datasets.	Caries detection, diagnostic imaging, orthodontic prediction, treatment planning.
Ferrara et al. (97)	Periodontology	DL, CNN, U-Net, YOLO, Mask R-CNN, GAN, Random Forest (RF), Support Vector Machine (SVM), CAD systems	Limited generalizability, heterogeneous datasets.	Periodontal diagnosis, alveolar bone loss detection, periodontitis staging, image segmentation.
Haj et al. (98)	Gerodontology/Oral Health in Older Adults	Teledentistry, mobile health (mHealth) applications, web-based platforms, social media, VR, augmented reality (AR)	Low digital literacy, technical limitations.	Oral health education, prevention, remote monitoring.
Kaushik, et al. (99)	Teledentistry	AI, automated image analysis, remote patient monitoring, Internet of Things (IoT), extended reality (XR), 5G, blockchain	Limited digital infrastructure, privacy and cybersecurity concerns	Remote diagnosis, treatment planning, patient monitoring.
Alfaraj et al. (100)	Prosthodontics and Implant Dentistry	AI, ML, DL, Artificial Neural Networks (ANNs), NLP, robotics, genetic algorithms, implant classification models, prosthesis design software, predictive models	Limited data diversity, poor model generalizability	Diagnostic imaging, implant classification, treatment planning, prosthesis design.
Albalawi et al. (102)	Orthodontics	LLMs, ChatGPT, Google Bard/Gemini, Microsoft Bing/Copilot AI, DISCERN assessment tool, Likert-scale evaluation	High risk of bias due to lack of standardized evaluation tools	Orthodontic education, answering orthodontic-related queries, patient information.
Mahendra et al. (104)	Periodontology	ChatGPT, AI, NLP, LLMs, ML, DL	Lack of empirical evidence in periodontology	Clinical decision support, patient education, disease risk assessment.
Dettori et al. (105)	General Oral Health Care Sciences	AI, ML, DL, Diagnostic imaging systems, Decision-support systems, Teledentistry	Ethical and regulatory challenges, transparency and explainability	Diagnostic imaging, caries detection, prosthodontic design, decision support.
Baxmann, et al. (106)	Dental and Orthodontic Education	Generative AI, LLMs, ChatGPT, ML, Adaptive learning platforms, AI tutoring systems, Clinical simulation tools, Real-time feedback systems	Limited faculty training	Knowledge retention, clinical decision-making training, personalized learning.
Lombardi, et al. (101)	Digital Implantology and Implant Prosthodontics	AI, ML, DL, rule-based AI, robotics, computer-assisted surgery, CBCT, multimodal data registration, VR, digital smile design.	Interoperability issues, lack of standardization, limited clinical validation	Diagnostic imaging, implant planning, prosthetic design, digital smile design.
de Magalhães et al. (107)	Dental Diagnostic Imaging	Cone Beam Computed Tomography (CBCT), AI, AI-driven diagnostic algorithms, biosensors, molecular diagnostics, advanced digital imaging technologies	High implementation costs	Early diagnosis, diagnostic imaging, periodontal disease detection, dental fracture detection.
Ardila et al. (103)	Orthodontic Education	AI, ChatGPT, LLMs, AI-assisted cephalometric landmarking platforms	Limited curricular integration	Orthodontic education, cephalometric analysis training, diagnostic skill development.
Shujaat (109)	General Dentistry	AutoML, ML, DL, Google Cloud AutoML, H2O Driverless AI, Neural Networks, Hyperparameter Optimization, Feature Engineering, Automated Model Selection	Limited high-quality datasets	Automated diagnosis, image analysis, disease prediction, treatment planning.
Shneiderman, et al. (110)	General Dentistry (Clinical Applications of AI)	AI, ML, DL, Computer Vision, Diagnostic Algorithms, Clinical Decision Support Systems, Predictive Models	Limited evidence quality	Image classification, diagnosis and detection, prognosis prediction.
Khattak et al. (108)	Dental Image-Based Diagnostics	DL, Convolutional Neural Networks (CNNs), ANNs, ML, AI diagnostic models, CAD/CAM-assisted systems, robotic-assisted procedures	Limited and biased datasets, high implementation costs, data privacy and security concerns	Caries detection, apical lesion detection, periodontal bone loss assessment, image segmentation.
Monalisa et al. (111)	Digital Dentistry, Dental Education	Additive Manufacturing (AM), 3D Printing, CAD/CAM, intraoral scanners, SLA, DLP, FDM, SLS, PolyJet, biocompatible materials	High initial investment, material toxicity concerns, regulatory barriers	Fabrication of crowns, bridges, dentures, surgical guides, orthodontic appliances.
Bors et al. (112)	Prosthodontics	CAD/CAM, digital impressions, intraoral scanners, 3D printing (additive manufacturing), AI, embedded microsensors, smart dentures, regenerative biomaterials	High implementation costs, limited clinical evidence	Digital denture design and fabrication, complete denture rehabilitation.
Samaranayake et al. (113)	General Dentistry	AI, ML, DL, NLP, Computer Vision, LLMs, Generative AI, Clinical Decision Support Systems (CDSS), diagnostic imaging AI	Ethical and legal concerns, data privacy and security	Diagnostic imaging, disease detection, risk prediction, treatment planning, clinical decision support.
Hassani et al. (114)	Implant Dentistry, Periodontology, and Oral Surgery	AI, ML, DL, predictive modeling, diagnostic algorithms, nanotechnology, nanomaterials.	Ethical concerns, data privacy and security	Implant planning, diagnostic support, outcome prediction, periodontal assessment.
Ardila, et al. (115)	Endodontic Education	AI, LLMs, CNNs, diagnostic AI systems, educational assessment tools.	Limited number of student-based studies	Endodontic diagnosis training, formative tutoring.
Our Study	Entire dental curriculum	ML, DL, CNN, ChatGPT, GPT-4o, Gemini, Claude, Microsoft Copilot, DeepSeek, RAG assistants, prompt engineering, computer vision, predictive analytics	Methodological heterogeneity across study designs, educational settings, AI models, and outcome measures.	Diagnostic support; personalized tutoring.

AM, additive manufacturing; ANN, artificial neural network; AutoML, automated machine learning; CAD/CAM, computer-aided design/computer-aided manufacturing; CDSS, clinical decision support system; DL, deep learning; DLP, digital light processing; FDM, fused deposition modeling; ML, machine learning; SLA, stereolithography; SLS, selective laser sintering; VR, virtual reality.

**SCHEME 1:**
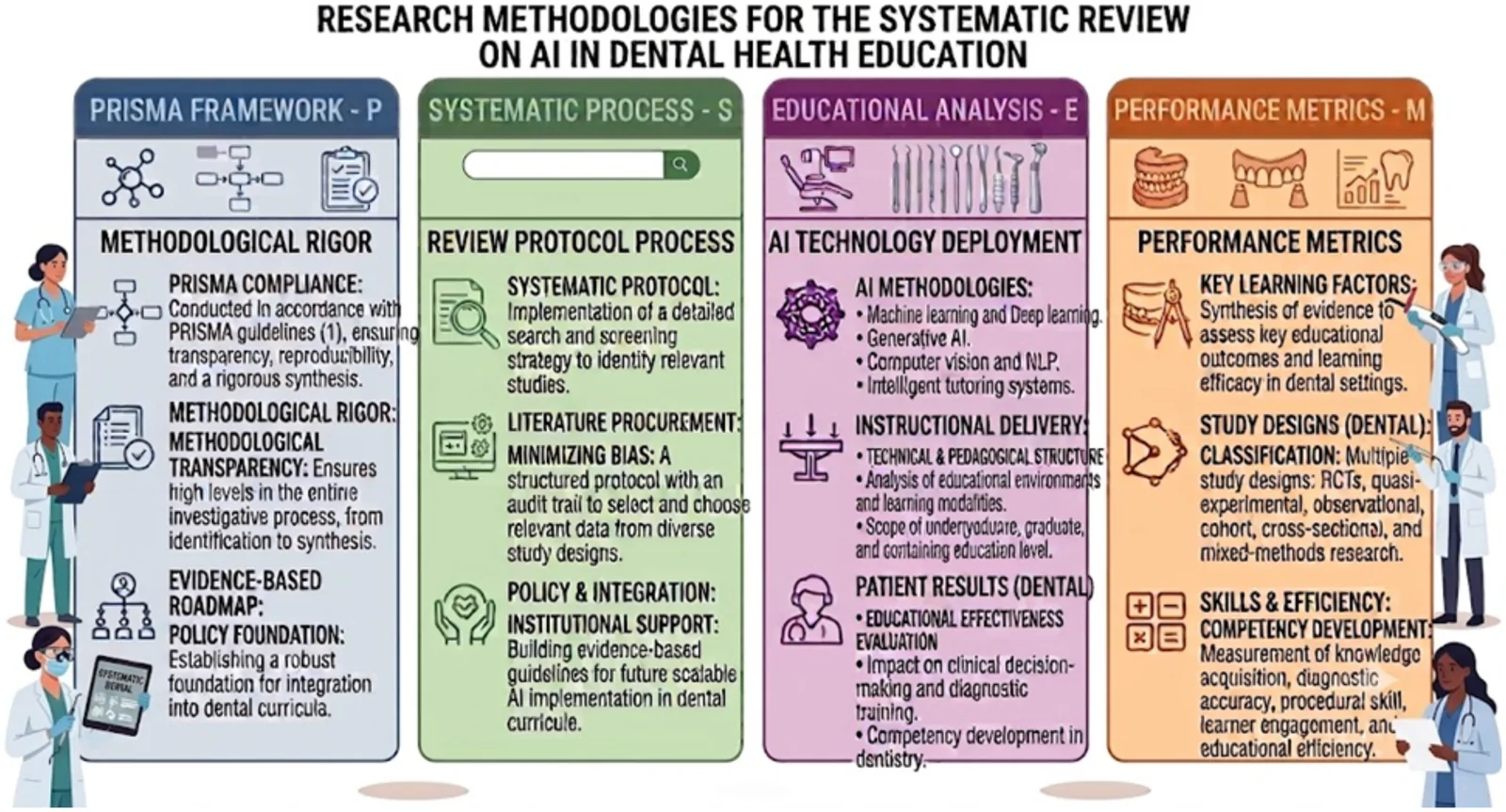
Advancing Odontology Education through Artificial Intelligence: Integrating Innovation, Research, and Evidence-Based Pedagogical Transformation.

**SCHEME 2:**
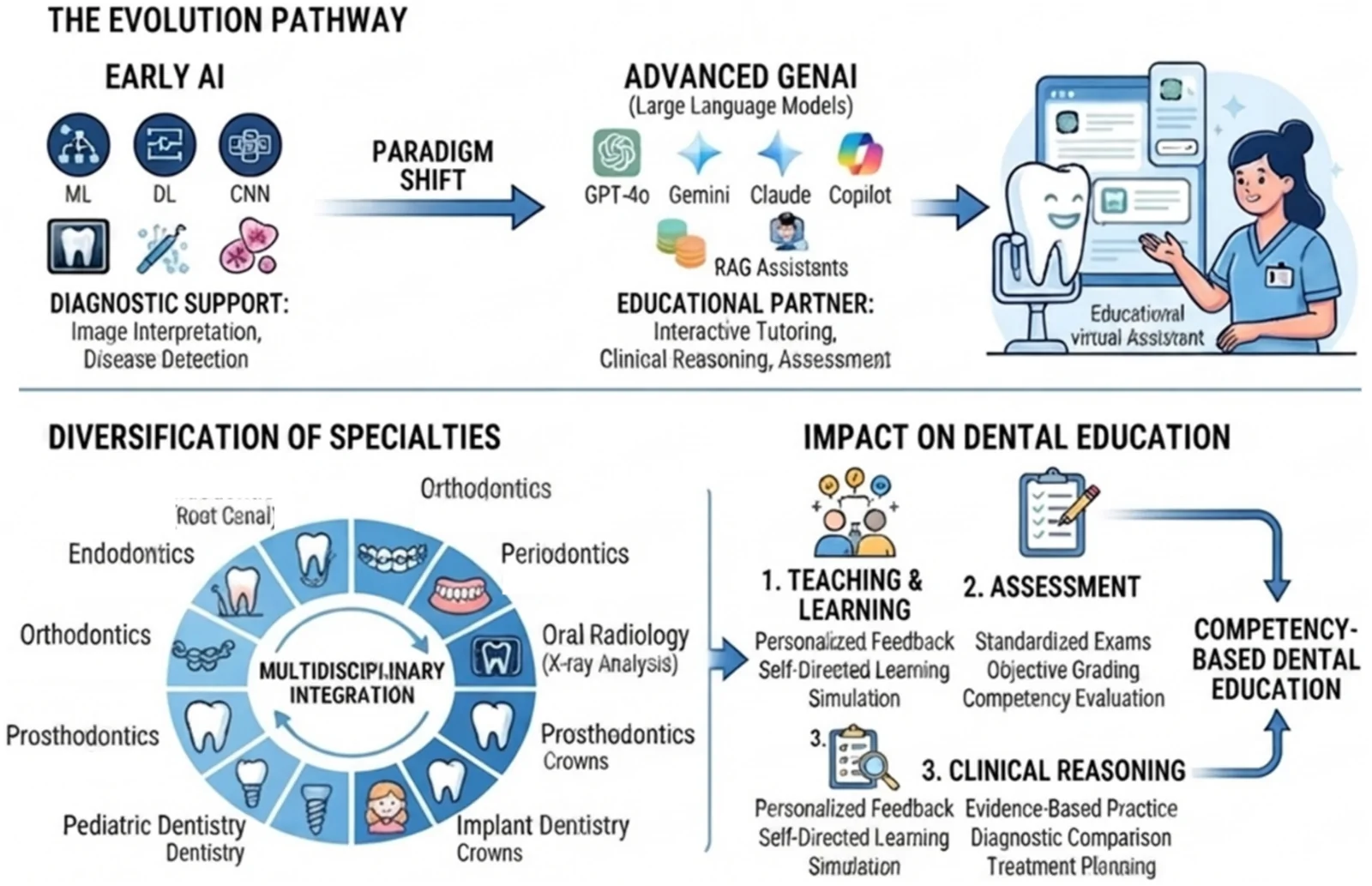
Evolution of AI and its educational applications in dental education.

**SCHEME 3:**
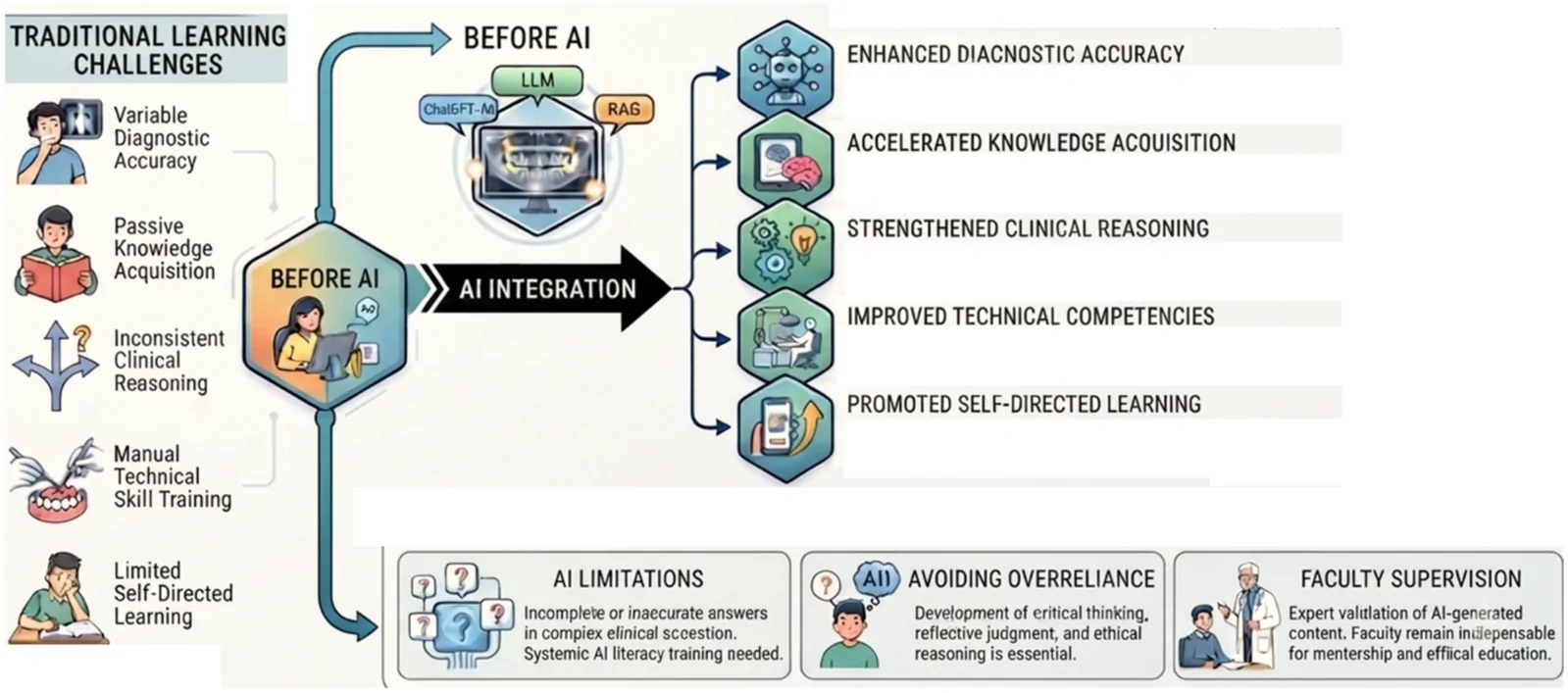
Impact of AI integration on learning outcomes and student performance in dental education.

The Table 6 presents the documented expansion of AI across dentistry, but most examined restricted specialties, technologies, or clinical applications. Pediatric and special care dentistry reviews emphasized caries detection, behavior management, teledentistry, and monitoring, while noting limited pediatric datasets and regulatory constraints (96). Periodontology-focused syntheses concentrated on deep learning for bone-loss detection, staging, segmentation, and prognosis (97), whereas gerodontology and teledentistry reviews addressed remote education, access, monitoring, and digital-literacy barriers (98, 99). Evidence from prosthodontics and implant dentistry highlighted predictive modeling, prosthesis design, implant classification, and workflow optimization (100, 101). Orthodontic reviews evaluated LLMs, cephalometric training, learning, and educational content, but reported heterogeneous assessment methods and the need for expert oversight (102, 103). Other reviews examined ChatGPT in periodontology (104), clinical and multimodal applications (105), AI-supported dental education (106), diagnostic imaging (107, 108), automated machine learning (109), and clinical decision support (110, 111).

Against this fragmented background, our study stands out by integrating the full technological continuum—from ML, DL, and CNN-based systems to generative AI, multimodal LLMs, and RAG-assisted platforms—within a unified educational framework. Rather than limiting analysis to one specialty or clinical function, it synthesizes empirical evidence across undergraduate, postgraduate, and continuing education and evaluates knowledge acquisition, diagnostic accuracy, clinical reasoning, technical competence, learner engagement, assessment, and self-directed learning. It extends prior literature by connecting technological maturity with pedagogical outcomes and implementation stages.

### 4.4 Limitations

Many studies evaluated AI performance under controlled conditions using small, single-center samples or short-term assessments. Considerable variability was observed in study designs, learner populations, educational settings, AI models, and outcome measures, limiting direct comparison across investigations. Most studies focused on diagnostic accuracy, examination performance, or user perceptions, whereas long-term effects on knowledge retention, clinical competence, and patient care remain insufficiently explored. Because the evidence base was dominated by observational, cross-sectional, and technical performance studies, the reported associations should not be interpreted as demonstrating causal improvements in educational outcomes. The rapid updates to AI platforms may influence the reproducibility of reported results.

## 5 Conclusion

This systematic review indicates growing investigation and application of AI technologies across areas relevant to dental education, including teaching, assessment, diagnostic training, clinical reasoning, and competency development. The literature reflects a technological transition from machine learning and computer vision approaches toward generative AI, large language models, and retrieval-augmented systems, which are increasingly being evaluated as potential complementary educational resources. Across dental specialties, many studies reported favorable findings in diagnostic performance, examination-based assessments, selected skills, learner perceptions, and AI-assisted learning activities. However, these findings should be interpreted cautiously because much of the available evidence derives from technical performance assessments, cross-sectional studies, short-term evaluations, or indirect educational outcomes. Evidence regarding long-term knowledge retention, clinical competence, and educational effectiveness remains limited.

The current evidence therefore suggests that AI may complement selected aspects of dental education when used with faculty supervision, critical appraisal, and appropriate ethical safeguards. Further prospective, comparative, and longitudinal studies using standardized educational outcomes are needed before broader conclusions regarding curricular effectiveness or widespread integration can be established.

Accordingly, favorable technical or examination performance should not be equated with broad educational improvement, particularly given the limited evidence concerning skill development, learner engagement, long-term knowledge retention, and clinical competence.

## Data Availability

The original contributions presented in the study are included in the article/Supplementary Material, further inquiries can be directed to the corresponding authors.
