# Supplementary material for: Artificial intelligence in odontology education
Source: Front Digit Health. 2026 Oct 27;8:1965185. doi: 10.3389/fdgth.2026.1965185 (PMC13748459; doi:10.3389/fdgth.2026.1965185)
Supplement: Supplementary file 1 [file Supplementaryfile1.docx]

**SUPPLEMENTARY INFORMATION:**

**Artificial Intelligence in Odontology Education**

María Belén Morales-Cevallos1,*, Canva Byron Ma Lam2,*, Kathy Violeta Serrano Avalos3, Mónica Jimena Concha Guailla3, Diego Fabian Vique Lopez4

1 Universidad Ecotec, Km. 13.5 Samborondón, Samborondón, EC092302, Ecuador

2 Universidad Católica Santiago de Guayaquil, Guayaquil, Ecuador

3 Facultad de Salud Pública, Escuela Superior Politécnica de Chimborazo (ESPOCH), Riobamba, Ecuador

4 Italian University Institute of Rosario (IUNIR), Rosario, Argentina

* Correspondence: mmoralesc@ecotec.edu.ec (M.B.M-C) & canva.ma@cu.ucsg.edu.ec (C.B.M.L.)

**Prompt Scheme 1**

Create a high-quality scientific infographic in a professional flat vector style for an academic journal. The infographic should have a clean white background and be divided into four vertical rounded panels with soft pastel colors (blue, green, purple, and orange). At the top, include the title: "Research Methodologies for the Systematic Review on Artificial Intelligence in Dental Health Education". Panel 1 (blue), titled "PRISMA Framework", should illustrate methodological rigor, PRISMA compliance, literature identification, screening, transparency, reproducibility, evidence synthesis, and policy foundation using icons such as researchers, clipboards, flowcharts, checklists, magnifying glasses, and scientific diagrams. Panel 2 (green), titled "Systematic Process", should depict the review protocol, database searching, literature procurement, study selection, inclusion and exclusion criteria, bias minimization, policy integration, and evidence-based curriculum development using search bars, documents, databases, workflow arrows, and organizational icons. Panel 3 (purple), titled "Educational Analysis", should illustrate AI technologies applied to dental education, including Machine Learning, Deep Learning, Computer Vision, Natural Language Processing, Generative AI, Large Language Models, Intelligent Tutoring Systems, Retrieval-Augmented Generation, adaptive learning, simulation, clinical decision-making, competency development, and educational effectiveness using neural network icons, AI brains, teeth, dental instruments, digital interfaces, graphs, and educational symbols. Panel 4 (orange), titled "Performance Metrics", should present educational outcomes including diagnostic accuracy, clinical reasoning, learner engagement, procedural skills, competency-based education, academic performance, educational efficiency, assessment methods, and evidence synthesis using charts, dental illustrations, students, evaluation icons, and performance indicators. Maintain a modern editorial infographic style similar to figures published in Frontiers, Nature, or Elsevier journals, with Adobe Illustrator-quality vector graphics, consistent iconography, balanced spacing, subtle shadows, rounded corners, minimalist typography, high visual hierarchy, publication-ready quality, ultra-detailed medical illustration, clean scientific communication, professional academic design, 4K resolution, vector infographic, BioRender-inspired aesthetics, without logos or watermarks.

**Prompt Scheme 2**

Create a high-quality scientific infographic in a clean flat vector style for an academic journal on Artificial Intelligence in Dental Education. Use a white background, modern editorial design, soft blue color palette with subtle pastel accents, rounded icons, clean typography, and publication-ready vector graphics. Organize the infographic into four connected sections. At the top left, create a section titled "The Evolution Pathway" showing the progression from Early AI (Machine Learning, Deep Learning, Computer Vision, CNN) used for diagnostic support (image interpretation, disease detection) to Advanced Generative AI (GPT-4o, Gemini, Claude, Copilot, Retrieval-Augmented Generation assistants) connected by a large arrow labeled "Paradigm Shift", emphasizing the transition toward educational partners that provide interactive tutoring, clinical reasoning, assessment, and personalized learning. On the top right, illustrate a modern digital educational environment with a virtual AI assistant displayed on a computer screen, a tooth icon, and a dental educator interacting with the system. At the bottom left, create a circular multidisciplinary wheel titled "Diversification of Specialties", showing AI integration across Endodontics, Orthodontics, Periodontics, Prosthodontics, Pediatric Dentistry, Implant Dentistry, Oral Radiology, Oral Surgery, Restorative Dentistry, and Oral Pathology using dental icons arranged around the wheel with the center labeled "Multidisciplinary Integration". On the bottom right, create a section titled "Impact on Dental Education" divided into three major components: (1) Teaching and Learning with personalized feedback, adaptive learning, simulation, and self-directed learning; (2) Assessment with objective grading, competency evaluation, standardized examinations, automated feedback, and performance analytics; and (3) Clinical Reasoning with evidence-based practice, diagnostic reasoning, treatment planning, decision support, and case-based learning. Connect these three components with arrows leading to a final box labeled "Competency-Based Dental Education". Include professional medical icons, AI symbols, neural networks, dental instruments, digital dashboards, educational graphics, and workflow arrows. Use Adobe Illustrator-quality vector graphics, BioRender-inspired scientific illustration, balanced spacing, minimalistic design, consistent iconography, subtle gradients, soft shadows, and an elegant layout suitable for publication in Frontiers, Nature, or Elsevier journals. Ultra-high resolution, 4K, publication-ready, no logos, no watermarks, no unnecessary decorative elements.

**Prompt Scheme 3**

Create a professional scientific infographic in a modern flat vector style illustrating the transformation of dental education through Artificial Intelligence. Use a clean white background with soft blue and teal accents, rounded boxes, subtle gradients, thin connecting arrows, and publication-quality vector graphics suitable for Frontiers, Nature, or Elsevier journals. Organize the infographic into three horizontal sections. On the left, create a vertical panel titled "Traditional Learning Challenges" with illustrated icons representing: Variable Diagnostic Accuracy, Passive Knowledge Acquisition, Inconsistent Clinical Reasoning, Manual Technical Skill Training, and Limited Self-Directed Learning. In the center, design a transition pathway beginning with a hexagonal box labeled "Before AI", followed by a large black arrow labeled "AI Integration". Above the arrow, illustrate modern AI technologies including Large Language Models (LLMs), ChatGPT-4o, Gemini, Claude, Copilot, Retrieval-Augmented Generation (RAG), machine learning, deep learning, computer vision, and intelligent tutoring systems displayed around a computer monitor showing dental radiographic analysis. On the right, connect the AI integration arrow to five vertically aligned hexagonal icons representing the educational outcomes: Enhanced Diagnostic Accuracy, Accelerated Knowledge Acquisition, Strengthened Clinical Reasoning, Improved Technical Competencies, and Promoted Self-Directed Learning. Each outcome should have a clean icon (brain, microscope, neural network, dental instruments, digital learning, clinical decision-making) connected with thin blue arrows. At the bottom, include three rounded rectangular information boxes titled "AI Limitations", "Avoiding Overreliance", and "Faculty Supervision". Under AI Limitations, illustrate incomplete or inaccurate answers, hallucinations, algorithm bias, and the need for AI literacy. Under Avoiding Overreliance, emphasize critical thinking, reflective judgment, ethical reasoning, and independent decision-making. Under Faculty Supervision, illustrate educators mentoring students while validating AI-generated content, emphasizing human oversight, evidence-based teaching, and responsible AI use. Include professional medical and dental icons, AI symbols, neural networks, digital dashboards, radiographs, teeth, educational illustrations, workflow arrows, and minimalist typography. Use Adobe Illustrator-quality vector graphics, BioRender-inspired scientific illustration, balanced composition, consistent iconography, soft shadows, subtle pastel colors, high visual hierarchy, ultra-high resolution (4K), publication-ready quality, and no logos or watermarks
